# Perspective on Many-Body Methods for Molecular Polaritonic Systems

**DOI:** 10.1021/acs.jctc.5c00801

**Published:** 2025-10-17

**Authors:** Nicholas Bauman, Leonardo A. Cunha, A. Eugene DePrince, Johannes Flick, Jonathan J. Foley, Niranjan Govind, Gerrit Groenhof, Norah Hoffmann, Karol Kowalski, Xiaosong Li, Marcus Liebenthal, Neepa T. Maitra, Ruby Manderna, Mikuláš Matoušek, Ilia M. Mazin, Daniel Mejia-Rodriguez, Ajay Panyala, Bo Peng, Benjamin Peyton, Libor Veis, Nam Vu, Jared D. Weidman, Angela K. Wilson, Rhiannon A. Zarotiadis, Yu Zhang

**Affiliations:** † Physical Sciences Division, 6865 Pacific Northwest National Laboratory, Richland, Washington 99352, United States; ‡ Center for Computational Quantum Physics, 525571 Flatiron Institute, New York, New York 10010, United States; § Department of Chemistry and Biochemistry, 7823 Florida State University, Tallahassee, Florida 32306-4390, United States; ∥ Department of Physics, 14770 City College of New York, New York, New York 10031, United States; ⊥ Department of Chemistry, 2331 University of North Carolina Charlotte, Charlotte, North Carolina 28223, United States; # Department of Chemistry, University of Washington, Seattle, Washington 98195, United States; ∇ Nanoscience Center and Department of Chemistry, 205518 University of Jyvaskyla, Jyvaskyla 40014, Finland; ○ Department of Chemistry and Physics, 5894 New York University, New York, New York 10003, United States; ◆ Department of Physics, University of Washington, Seattle, Washington 98195, United States; ¶ Department of Physics, 7284 Rutgers University, Newark, New Jersey 07102, United States; ⋈ J. Heyrovský Institute of Physical Chemistry, Academy of Sciences of the Czech Republic, v.v.i., Dolejškova 3, 18223 Prague 8, Czech Republic; ⧓ Faculty of Mathematics and Physics, 5924 Charles University, 12116 Prague 2, Czech Republic; ⧖ Theoretical Division, 14727 Los Alamos National Laboratory, Los Alamos, New Mexico 87544, United States; ● Advanced Computing, Mathematics and Data Division, 86875 Pacific Northwest National Laboratory, Richland, Washington 99354, United States; ▲ Department of Chemistry, 5112 Michigan State University, East Lansing, Michigan 48824, United States; ■ Simons Center for Computational Physical Chemistry, New York University, New York, New York 10003, United States

## Abstract

Recent advances in strong light–matter interactions have revealed a wealth of new physical phenomena in molecules embedded in optical cavities, including modified chemical reactivity, altered excitation spectra, and novel quantum correlations. To describe these effects from first-principles, the field of *ab initio* quantum electrodynamics (QED) has emerged as a compelling extension of quantum chemistry that treats electronic and photonic degrees of freedom on equal footing. In this Perspective, we review the growing landscape of many-body QED methods, including Hartree–Fock, density functional theory (QEDFT), time-dependent DFT (QED-TDDFT), configuration interaction (QED-CI), complete active space (QED-CASSCF), coupled cluster (QED-CC), quantum Monte Carlo (QED-QMC), and density matrix renormalization group (QED-DMRG), highlighting recent developments and implementations. We further explore real-time methods, gradient and Hessian formalisms, and the integration of nonadiabatic nuclear dynamics. Applications range from benchmark simulations of polaritonic chemistry to quantum simulations on emerging quantum hardware. We conclude by outlining future directions for theory development and interdisciplinary efforts at the interface of quantum chemistry, condensed matter, and quantum optics.

## Graphical abstract

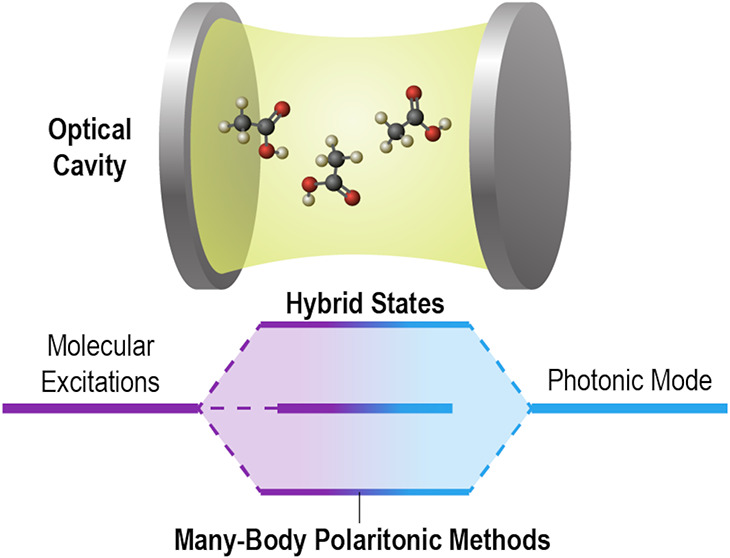

## 1 Introduction

Placing a photoactive material inside an optical microcavity or near a plasmonic nanoresonator can lead to measurable changes in its properties and, consequently, alter its propensity for energy transfer,^1–8^ charge transport,^9–12^ lasing,^13–15^ or even its photochemistry.^16–20^ By restricting the number of available optical modes and confining the electromagnetic field to small volumes, micro- and nanoresonators enhance the light–matter interaction strength.^21^ When this interaction exceeds the intrinsic losses of both the photoactive material and the resonator, excitons and confined light modes hybridize to form new light–matter states known as polaritons.^22–25^ With low effective masses and high group velocities from their photonic component, and large interaction cross sections from their matter component, polaritons are attractive quasiparticles for applications such as Bose–Einstein condensation and energy transport.

However, due to the inherent complexity of organic materials, it remains unclear how the formation of these polaritonic states leads to the experimentally observed modifications in material properties.^26^ For instance, the role and nature of dark states in many-molecule strong coupling, particularly how they depend on cavity parameters, solvent, and molecular configurations, remains unresolved. These states are predominantly (though not entirely^27–29^ ) localized excitations with little photonic character. Even if they are largely unaffected by the light–matter coupling itself, their interactions with polaritonic states that lie nearby in energy can cause ultrafast decoherence throughout the system. Yet the problem is subtle,^30–32^ and observed lifetimes are often longer than expected.

Furthermore, a recent transient absorption/reflection spectroscopy study has called for a careful reevaluation of several effects previously attributed to polariton formation, suggesting they may instead arise from nontargeted consequences of photoexcitation.^33^ Another open question concerns the interplay between collective and local interactions. While collective strong coupling involves a large ensemble of molecules, it can still induce strong local modifications to the potential energy landscape, effectively giving rise to local strong coupling.^34,35^

As such, the theoretical community has been actively developing new frameworks that integrate quantum optics into quantum chemistry, aiming to build predictive models for the effects of strong light–matter coupling on molecular properties. For quantitative and realistic descriptions, it is necessary to go beyond the standard few-level, single-mode models traditionally used in cavity quantum electrodynamics (QED),^36–38^ whose simplifying assumptions often break down in real experimental systems.^26^ These systems involve real molecules, complex environments, and cavities that support a continuum of modes and exhibit losses.^39–46^ Consequently, the development of many-body QED methods, which treat electrons and photons on equal footing, has gained significant traction in the quantum chemistry community.^26,40,47,48^

A many-body QED approach requires careful identification of the physical quantities (Figure 1) that govern light–matter interactions. Naturally, we begin with the same foundational elements as in traditional quantum chemistry, such as the one- and two-electron integrals. To this, we add essential cavity parameters: the cavity mode frequencies (ω), their polarizations (**ê**), and the mode volume (*V*). The interaction between the molecules and the cavity is mediated by the effective dipole moment, assuming no disorder (all molecules are aligned), can be defined as $\mu_{\mathrm{eff}}=\sqrt{N}\mu$ , where *N* is the number of molecules and μ is the dipole moment of a single molecule. The light–matter interaction energy scales as $g\propto \sqrt{\omega }\left(\lambda \cdot \mu_{\mathrm{eff}}\right)$ , where $\lambda \propto \sqrt{4\pi /V}ê$ characterizes the field strength along the polarization axis in volume *V*. For cavities supporting multiple modes with the same polarization, the effective coupling can be generalized as $\lambda_{\mathrm{eff}}^{2}=\overset{M}{\underset{a=1}{\sum }}\lambda_{a}^{2}$ . Additional quantities, such as cavity losses for each mode, may also be considered, but the parameters described above are universal in the QED literature.

**Figure 1:**
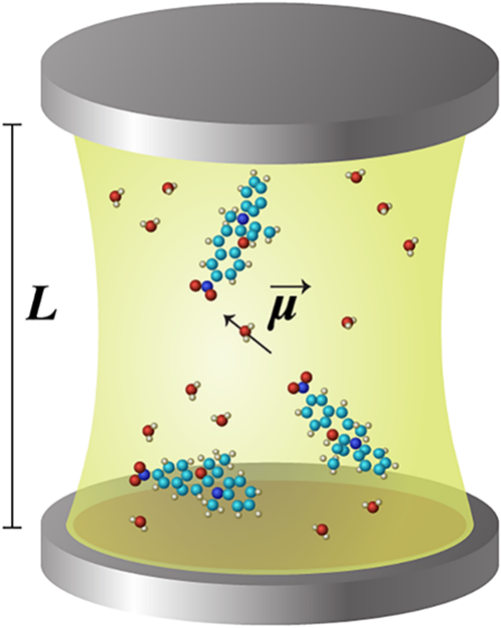
Schematic of a cavity–molecule system highlighting key physical parameters that govern the light–matter interaction strength. The coupling scale *g* depends on the cavity mode frequency ω, mode volume *V*, and the collective dipole moment $\mu_{\mathrm{eff}}=\sqrt{N}\mu$ of the molecular ensemble.

In this Perspective, we survey recent developments in many-body QED methods for quantum chemistry, with an emphasis on how electronic strong coupling and exciton–polariton formation are described across different theoretical levels. We begin with the Pauli–Fierz Hamiltonian, which serves as the foundation for all first-principles QED approaches, and use it to introduce a range of formalisms, including QED Hartree–Fock (QED-HF),^49–52^ QED density functional theory (QEDFT),^53–55^ time-dependent QEDFT (QED-TDDFT),^56,57^ QED configuration interaction (QED-CI),^47,58,59^ QED complete active space self-consistent field (QED-CASSCF),^60,61^ and QED coupled cluster (QED-CC) methods.^49,62,63^ We also describe extensions to quantum Monte Carlo (QED-QMC)^64^ and density matrix renormalization group (QED-DMRG)^65^ approaches, which enable treatment of strong correlation and large active spaces in cavity-coupled systems. Several important developments have also been made within the framework of perturbation theory, including Møller–Plesset theory applied to the Pauli–Fierz Hamiltonian and in conjunction with the variational Lang-Firsov transformation,^66,67^ and in using algebraic diagramatic construction.^68^ Beyond static electronic structure, we discuss how QED frameworks incorporate nuclear motion, including recent work on analytic gradients, vibrational strong coupling, real-time dynamics, and the breakdown of the Born–Oppenheimer approximation under light–matter coupling. We conclude with an outlook on emerging opportunities at the intersection of QED, nonadiabatic dynamics, quantum simulation, and data-driven theory development, where continued progress will be essential to unravel the microscopic mechanisms by which quantum light modifies molecular structure and reactivity.

## 2 Pauli–Fierz Hamiltonian

Now that we have introduced the physical principles and challenges underlying polaritonic chemistry, we proceed to establish the core mathematical framework needed to model light–matter interactions within many-body QED methods. The traditional electronic Hamiltonian is extended to treat electronic and photonic degrees of freedom on equal footing. Additional terms account for photon energy and its coupling to matter.

The minimally coupled Coulomb Hamiltonian, which governs the nonrelativistic dynamics of matter in an electromagnetic (EM) environment, is^53,69–71^

1 $$Ĥ=\sum_{i}^{N_{e}+N_{n}}\frac{1}{2m_{i}}{\left[{\mathrm{p̂}}_{i}-\frac{z_{i}}{c}Â\left(r_{i}\right)\right]}^{2}+\mathrm{V̂}+{Ĥ}_{\mathrm{EM}}$$

where ***A* ^** is the magnetic vector potential, ${\mathrm{p̂}}_{i}$ is the momentum operator of the *i* ^th^ particle, and *z*_*i*_ its charge. The total number of particles includes both electrons (*N*_*e*_) and nuclei (*N*_*n*_). For electrons, the charge is given by the individual electron charge −*e*, and for nuclei, the charge is the total nuclear charge *Ze* where *Z* denotes the atomic number. The potential term $\mathrm{V̂}={\mathrm{V̂}}_{\mathrm{ee}}+{\mathrm{V̂}}_{\mathrm{nn}}+{\mathrm{V̂}}_{\mathrm{en}}+V_{\mathrm{ext}}$ contains all Coulomb interactions between electronic and nuclear degrees of freedom, along with any external potential that may be present.

Working within the Coulomb gauge, where ∇· ***A*** = 0, the Hamiltonian describing the electromagnetic field is given by

2 $${Ĥ}_{\mathrm{EM}}=\sum_{l=1}^{2}\int \hbar \omega_{k}{\mathrm{b̂}}^{†}\left(k,l\right)\mathrm{b̂}\left(k,l\right)dk$$

where *b̂* ^†^(**k**, *l*) and *b̂*(**k**, *l*) create and destroy a mode with wavevector **k** and polarization *l*, respectively. When matter is coupled to the full continuum of modes, then the masses in eq 1 are in fact the bare masses of the nuclei and electrons rather than the observable masses.^72^ For practical calculations on molecular systems in cavity environments, several approximations to eq 1 need to be made. First, rather than considering the full continuum of EM modes, one identifies only a small subset of (or even a single) relevant mode(s), which is equivalent to defining an energy cutt-off for eq 2 in *k*-space. Then, the discarded modes, which are assumed to be unaffected by the cavity, enter into the picture as the renormalized masses of the electrons and nuclei, leading to one using the observable masses of these particles in practical calculations (in the atomic unit system, the observable masses are *m*_*e*_ = 1 and *m*_*p*_ ≈ 1836).^73^ Unless otherwise noted, we assume coupling to a single mode in the following equations. Second, the long-wavelength or dipole approximation is typically invoked, which is based on the assumption that the variance of the fields/vector potential associated with the relevant mode(s) is negligible on the length scale of the matter subsystem. For a given mode within the long wavelength approximation, $Â\left(r_{i}\right)\approx A_{0}\sqrt{\frac{2\omega }{\hbar }}{\mathrm{x̂}}_{c}=A_{0}\left({\mathrm{b̂}}^{†}+\mathrm{b̂}\right)$ , where ω and *x̂*_c_ denote the frequency and canonical position operator for the mode, *b̂* ^†^ and *b̂* are the creation and annihilation operators for the mode, and $A_{0}=\sqrt{\frac{\hbar }{2\omega \epsilon_{0}V}}ê$ denotes the magnitude and direction of the vector potential of the mode dependent on the effective mode volume *V*. We have dropped the explicit notation for the wavevector and polarization of the modes for simplicity. We note that the field strength introduced earlier can be related to the vector potential magnitude as $A_{0}=\sqrt{\frac{1}{2\omega }}\lambda$ , and as vector quantities, **A**_0_ and **λ** point in the same direction. For molecular calculations, it is also common to transform Hamiltonian in eq 1 into length gauge using the Power–Zienau–Woolley (PZW) transformation followed by a second unitary phase transformation. The PZW operator has the form

3 $${Û}_{\mathrm{PZW}}=\exp\left[-\frac{i}{\hbar }\mathrm{\mu ̂}\cdot Â\right]$$

where **μ̂** is the total dipole operator (containing electronic and nuclear degrees of freedom), and Â is the vector potential operator within the long wavelength approximation. This yields the Pauli–Fierz Hamiltonian within the length gauge, which can be expressed as

$$Ĥ=\overset{N_{e}+N_{n}}{\underset{i}{\sum }}\frac{1}{2m_{i}}{\mathrm{p̂}}_{i}^{2}+\mathrm{V̂}+\hbar \omega {\mathrm{b̂}}^{†}\mathrm{b̂}-\sqrt{\frac{\omega }{2}}\lambda \cdot \mathrm{\mu ̂}\left({\mathrm{b̂}}^{†}+\mathrm{b̂}\right)+\frac{1}{2}{\left[\lambda \cdot \mathrm{\mu ̂}\right]}^{2}$$ 4

with the momentum, dipole, and potential operators still running over all electrons and nuclei as quantum particles. As remarked before, the electronic and nuclear masses in this single mode Hamiltonian are the observable masses rather than the bare masses used in eq 1. Finally, if the Born–Oppenheimer (clamped nuclei) approximation is invoked, we arrived at

5 $${Ĥ}_{\mathrm{PF}}={Ĥ}_{e}+\omega {\mathrm{b̂}}^{†}\mathrm{b̂}-\sqrt{\frac{\omega_{n}}{2}}\mathrm{d̂}\left({\mathrm{b̂}}^{†}+\mathrm{b̂}\right)+\frac{1}{2}{\mathrm{d̂}}^{2}$$

In eq 5, *Ĥ*_*e*_ denotes the standard molecular electronic Hamiltonian within the Born–Oppenheimer approximation. The term *ωb̂* ^†^ *b̂* represents the energy of the cavity (photonic) mode, where ω is the mode frequency and *b̂* ^†^, *b̂* are the bosonic creation and annihilation operators, respectively. The interaction between matter and field is captured by the bilinear coupling term and the dipole self-energy. Together, these account for both the coupling between photons and the molecular dipole and the cavity-mediated electron–electron correlation. The light–matter coupling is encoded in *d̂* = **λ**·**μ̂**, where **μ̂** = **μ̂**_el_ + **μ**_nuc_ contains an electronic dipole operator and a nuclear dipole moment that depends parametrically upon the nuclear charges and coordinates. These terms form the foundation for the many-body QED formalisms developed in recent theoretical work.

We note that invoking the Born–Oppenheimer approximation in ordinary quantum chemistry is based upon identify slow and fast coordinates when considering coupled electronic-nuclear many-body problems. Clearly, in eq 1 we are dealing with a coupled electronic-nuclear-photonic many-body problem, and the appropriate choice of slow and fast coordinates will depend upon the relevant energy scales. In some cases, it is advantageous to regard both the photonic and nuclear coordinates as the slow coordinates, while leaving the electrons as fast (quantum) degrees of freedom. Such a partitioning is referred to as the Cavity Born–Oppenheimer approximation (CBO),^74–80^ which we discuss in Section 14.

## 3 QED Hartree–Fock Theories

### 3.1 Direct Product Representations

A mean-field approach to *ab initio* QED Hamiltonians can be obtained through a modified Hartree–Fock procedure (QED-HF), wherein one writes the reference wave function as

6 $$\left|0^{e}0^{p}\right\rangle =\left|0^{e}\right\rangle ⊗\left|0^{p}\right\rangle$$

which is a direct product of a Slater determinant of electronic spin orbitals $\left(\left|0^{e}\right\rangle \right)$ and a mean-field photonic ground-state $\left(\left|0^{p}\right\rangle \right)$ . In the following discussion, our focus is primarily concerned with the single molecule limit of this ansatz, but we note that in the limit of a dilute ensemble of molecules in a cavity, one can write down an ensemble wave function for the molecular system as a product of Slater determinants.^81^ If one assumes each molecule to be identical, upon discarding the dipole self-energy and invoking a 2-level approximation for each molecular subsystem, the Tavis–Cummings model may be recovered (Figure 2).

Several formulations of QED-HF have been developed to generate the reference wave function, a brief overview of which is provided below.

**Figure 2:**
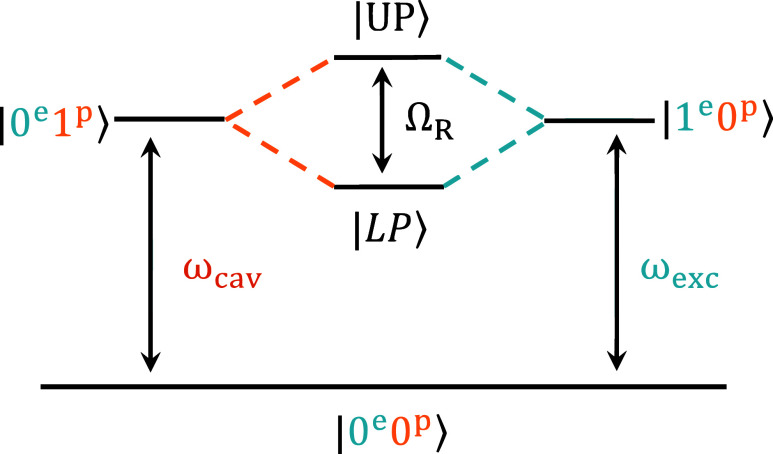
Model two-level diagram showing the coupling between $\left|0^{e}1^{p}\right\rangle$ (ground state with a photon at ω_cav_) and $\left|1^{e}0^{p}\right\rangle$ (electronic excited state at ω_exc_) into hybrid upper and lower polariton states (|UP⟩⁡and⁡|LP⟩) split by the Rabi frequency Ω_R_.

#### 3.1.1 Number State (Fock) Representation

The photonic wave function $\left|0^{p}\right\rangle$ can be expressed as a linear combination of photon number (Fock) states:

7 $$\left|0^{p}\right\rangle =\sum_{n}C_{n}{\left({\mathrm{b̂}}^{†}\right)}^{n}\left|0\right\rangle$$

where |0⟩ is the photon vacuum. The QED-HF wave function is then determined via a modified Roothaan–Hall procedure. In the first step, the electronic wave function $\left|0^{e}\right\rangle$ is optimized assuming a fixed zero-photon state. Given this electronic reference, the electronic degrees of freedom are integrated out of the Pauli–Fierz Hamiltonian to define an effective photonic Hamiltonian:

8 $${Ĥ}_{p}=\left\langle 0^{e}\right|{Ĥ}_{\mathrm{PF}}\left|0^{e}\right\rangle$$

whose lowest eigenfunction defines $\left|0^{p}\right\rangle$ . This two-step procedure is iterated until self-consistency is reached.

A critical detail in this representation is the convergence of the photon number basis. An incomplete photon basis can lead to spurious behavior. For instance, introducing a dependence of the QED-HF energy on the photon frequency ω_*n*_, or violating translational invariance in charged systems.^47^ These issues can be resolved by switching to the coherent-state representation described below.

#### 3.1.2 Coherent-State Representation

An alternative formulation of the QED-HF problem uses the coherent-state basis,^58,82^ which diagonalizes the photonic part of the Pauli–Fierz Hamiltonian. In this approach, a unitary displacement operator is applied to shift the photonic degrees of freedom by an amount proportional to the molecular dipole moment:

9 $${Û}_{\mathrm{CS}}=\exp\left[z\left({\mathrm{b̂}}^{†}-\mathrm{b̂}\right)\right]$$

where $z=-\left\langle \mathrm{d̂}\right\rangle /\sqrt{2\omega }$ for a cavity with a single photon mode of frequency ω.

The expectation value of the Hamiltonian in this displaced basis becomes

$$\left\langle 0^{e}0^{p}\right|{Ĥ}_{\mathrm{PF}}\left|0^{e}0^{p}\right\rangle =\left\langle 0^{e}\right|⊗\left\langle 0\right|{Û}_{\mathrm{CS}}{Ĥ}_{\mathrm{PF}}{Û}_{\mathrm{CS}}^{†}\left|0\right\rangle ⊗\left|0^{e}\right\rangle$$ 10

and the Pauli–Fierz Hamiltonian transforms into

11 $${Ĥ}_{\mathrm{CS}}={Ĥ}_{e}+\omega {\mathrm{b̂}}^{†}\mathrm{b̂}-\sqrt{\frac{\omega }{2}}\left({\mathrm{d̂}}_{e}-\left\langle {\mathrm{d̂}}_{e}\right\rangle \right)\left({\mathrm{b̂}}^{†}+\mathrm{b̂}\right)+\frac{1}{2}{\left({\mathrm{d̂}}_{e}-\left\langle {\mathrm{d̂}}_{e}\right\rangle \right)}^{2}$$

After the transformation, the nuclear dependence in the dipole operator vanishes, leaving only the electronic contribution *d̂*_e_. As a result, the photonic degrees of freedom no longer require self-consistent optimization. Instead, the light–matter coupling is encoded through the displacement parameter *z*.

The corresponding Fock matrix incorporates cavity-induced terms and is written as

12 $$F_{\mu \nu }=T_{\mu \nu }+V_{\mu \nu }+J_{\mu \nu }-K_{\mu \nu }+O_{\mu \nu }^{\mathrm{DSE}}+J_{\mu \nu }^{\mathrm{DSE}}-K_{\mu \nu }^{\mathrm{DSE}}$$

where *T*, *V*, *J*, and *K* are the usual kinetic, nuclear attraction, Coulomb, and exchange contributions. The remaining terms arise from the dipole self-energy (DSE) and are defined as

13 $$\begin{array}{l} O_{\mu \nu }^{\mathrm{DSE}}=\left\langle {\mathrm{d̂}}_{e}\right\rangle d_{\mu \nu }-\frac{1}{2}q_{\mu \nu }\\ J_{\mu \nu }^{\mathrm{DSE}}=\left\langle {\mathrm{d̂}}_{e}\right\rangle d_{\mu \nu }\\ K_{\mu \nu }^{\mathrm{DSE}}=\sum_{\lambda \sigma }d_{\mu \sigma }d_{\lambda \nu }\gamma_{\lambda \sigma } \end{array}$$

where *d*_μ_ν and *q*_μ_ν are field-weighted dipole and quadrupole integrals in the atomic orbital basis, and γ_λσ_ is the QED-HF density matrix. Similar cavity-induced terms appear in the QED-DFT framework as discussed in eq 30.

The coherent-state representation offers a variationally efficient route for modeling systems under weak to moderate light–matter coupling. However, as the coupling strength increases, more flexible and correlated ansätze are needed to capture photonic and electronic interactions beyond mean-field displacement.

### 3.2 Entangled Representations

#### 3.2.1 Strongly-Coupled QED-HF

The strongly coupled QED-HF (SC-QED-HF) method, developed by Riso and co-workers,^50,51^ generalizes the displacement operator used in the coherent-state formulation. Instead of relying on the expectation value of the molecular dipole, SC-QED-HF uses the full dipole operator **μ̂**, allowing for the treatment of light–matter interactions beyond mean-field effects.

The unitary transformation defining the method is

14 $${Û}_{\mathrm{SC}}=\exp\left[-\frac{\mathrm{d̂}}{\sqrt{2\omega }}\left({\mathrm{b̂}}^{†}-\mathrm{b̂}\right)\right]$$

where *d̂* = **λ**·**μ̂**. This transformation displaces the photonic field by an amount dependent on the instantaneous dipole operator rather than its average.

Applying this transformation, the Pauli–Fierz Hamiltonian becomes

15 $${Ĥ}_{\mathrm{PF}}^{\mathrm{SC}}={Ĥ}_{p}^{\mathrm{SC}}+{Û}_{\mathrm{SC}}{Ĥ}_{e}{Û}_{\mathrm{SC}}^{†}$$

where the photonic Hamiltonian in displaced coordinates is given by

16 $${Ĥ}_{p}^{\mathrm{SC}}=\omega \left({\mathrm{b̂}}^{†}-\frac{\mathrm{d̂}}{\sqrt{2\omega }}\right)\left(\mathrm{b̂}-\frac{\mathrm{d̂}}{\sqrt{2\omega }}\right)$$

The electronic Hamiltonian is likewise transformed. Using a second-quantized formulation, it becomes

17 $${Ĥ}_{e}^{\mathrm{SC}}=\sum_{\mathrm{pq}}h_{\mathrm{pq}}{â}_{p}^{†}{â}_{q}\exp\left[-\frac{\left(\eta_{\mathrm{pq}}\cdot \lambda \right)}{\sqrt{2\omega }}\left({\mathrm{b̂}}^{†}-\mathrm{b̂}\right)\right]+\frac{1}{2}\sum_{\mathrm{pqrs}}v_{\mathrm{pq}}^{\mathrm{rs}}{â}_{p}^{†}{â}_{r}^{†}{â}_{s}{â}_{q}\exp\left[-\frac{\left(\eta_{\mathrm{pq}}^{\mathrm{rs}}\cdot \lambda \right)}{\sqrt{2\omega }}\left({\mathrm{b̂}}^{†}-\mathrm{b̂}\right)\right]$$

where *h*_*pq*_ and *v*_*pq*_^*rs*^ are the one- and two-electron integrals, respectively, and the exponential terms arise from the dipole transformation in the molecular orbital basis. The orbital-dependent displacement parameters are given by

18 $$\eta_{\mathrm{pq}}=\eta_{p}-\eta_{q}$$

19 $$\eta_{\mathrm{pq}}^{\mathrm{rs}}=\eta_{p}+\eta_{r}-\eta_{s}-\eta_{q}$$

These parameters are treated as additional variational degrees of freedom and are optimized alongside the electronic wave function coefficients. This leads to a more flexible ansatz capable of describing strongly correlated electron–photon states.

SC-QED-HF thus extends the coherent-state formalism by explicitly incorporating electron–photon entanglement, enabling a more accurate description of polaritonic systems under strong coupling conditions.

#### 3.2.2 Variationally-Transformed QED-HF

The variationally transformed QED-HF (VT-QED-HF) method^52^ introduces an additional variational parameter *f*_cav_ that controls the extent of the photonic displacement applied to the wave function. This allows for a continuous interpolation between the standard QED-HF approach and the fully displaced SC-QED-HF framework.

The transformation is implemented via a unitary operator of the form:

20 $${Û}_{\mathrm{VT}}=\exp\left[-f_{\mathrm{cav}}\cdot \frac{\mathrm{d̂}}{\sqrt{2\omega }}\left({\mathrm{b̂}}^{†}-\mathrm{b̂}\right)\right]$$

where *f*_cav_ ∈ [0, 1] is treated as a variational parameter. When *f*_cav_ = 0, the transformation reduces to the identity (i.e., standard QED-HF), while *f*_cav_ = 1 recovers the fully displaced SC-QED-HF ansatz.

By optimizing *f*_cav_ alongside the electronic orbitals, VT-QED-HF identifies a reference wave function that minimizes the total energy of the coupled electron–photon system. This strategy allows for flexible treatment of systems spanning a broad range of coupling strengths.

A related approach was introduced by Cui and co-workers,^66^ who implemented a similar transformation but with certain constraints imposed on the displacement operator. In both cases, the variational control over displacement enables a more accurate description of light–matter hybridization effects, especially in regimes where mean-field or fully entangled limits are insufficient.

#### 3.2.3 Variationally-Squeezed QED-HF

The variationally squeezed QED-HF (VSQ-QED-HF) method extends previous QED-HF frameworks by incorporating both photonic displacement and squeezing transformations. This approach allows for variational control over quantum fluctuations in the photonic field, enabling a more flexible description of electron–photon correlation.

Two key operators define the ansatz. The displacement operator *D̂*(*z*) = ∏_*n*_ *e* ^–(*z*_*n*_ *b̂*_*n*_–h.c.)^ generates a coherent state |*z*⟩ from the vacuum, where $z_{n}=\lambda_{n}\cdot \left\langle D\right\rangle /\sqrt{2\omega_{n}}$ represents the light–matter displacement. The squeezing operator is given by

21 $$Ŝ\left(F\right)=\prod_{n}\exp\left[\frac{1}{2}\left(F_{n}^{*}{\mathrm{b̂}}_{n}^{2}-F_{n}{\mathrm{b̂}}_{n}^{†2}\right)\right]$$

where *F*_*n*_ = *r*_*n*_ *e* ^*iθ*_*n*_^ encodes the squeezing amplitude and phase for each mode.^83,84^ In typical applications, $F_{n}\in R$ due to the real-valued nature of the basis and absence of gauge phases.

The full VSQ-QED-HF ansatz is defined as

22 $$\left|\Psi \right\rangle =\mathrm{D̂}\left(\mathrm{f̂}\right)Ŝ\left(F\right)\left|\mathrm{HF}\right\rangle ⊗\left|0_{p}\right\rangle \equiv Û\left(\mathrm{f̂},F\right)\left|\mathrm{HF}\right\rangle ⊗\left|0_{p}\right\rangle$$

where ${\mathrm{f̂}}_{n}=f_{n}\lambda_{n}\cdot D/\sqrt{2\omega_{n}}$ introduces variational flexibility in the displacement. Since *D̂* and *Ŝ* do not commute, different orderings lead to distinct variational forms, each capturing electron-boson correlations to different extents.^85^

Applying the combined transformation to the Pauli–Fierz Hamiltonian yields a modified operator:

23 $${Ĥ}_{\mathrm{CS}}={Ĥ}_{e}\left(\mathrm{X̂}\right)+\sum_{n}e^{-r_{n}}\sqrt{\frac{\omega_{n}}{2}}\Delta \lambda_{n}e_{n}\cdot D\left({\mathrm{b̂}}_{n}^{†}+{\mathrm{b̂}}_{n}\right)+\frac{1}{2}{\left(\Delta \lambda_{n}\right)}^{2}(e_{n}\cdot D{)}^{2}+{Ĥ}_{\mathrm{ph}}$$

where Δ*λ*_*n*_ = (1 – *f*_*n*_)**λ**_*n*_ and the dressed photonic Hamiltonian is given by

24 $${Ĥ}_{\mathrm{ph}}=\omega_{n}\left[\cosh\left(2r_{n}\right)\left({\mathrm{b̂}}_{n}^{†}{\mathrm{b̂}}_{n}+\frac{1}{2}\right)-\sinh\left(2r_{n}\right)\left({\mathrm{b̂}}_{n}^{2}+{\mathrm{b̂}}_{n}^{†2}\right)\right]$$

The total energy functional for VSQ-QED-HF becomes

25 $$E_{\mathrm{tot}}=\langle {Ĥ}_{e}\left(\mathrm{X̂}\right)\rangle_{\mathrm{HF}}+\frac{{\left(\Delta \lambda_{n}\right)}^{2}}{2}\left\langle \mathrm{HF}\right|(e_{n}\cdot \mathrm{D̂}{)}^{2}\left|\mathrm{HF}\right\rangle +\omega_{n}\cosh\left(2r_{n}\right)\left(n_{n}+\frac{1}{2}\right)$$

where the electronic energy functional resembles that of Hartree–Fock, but with integrals renormalized by the squeezing and displacement parameters.

Since cosh­(2*r*_*n*_) ≥ 1, the squeezed vacuum preserves the zero-point energy of the photonic field while allowing the energy surface to adjust variationally through the parameters *f*_*n*_ and *r*_*n*_. Unlike VT-QED-HF, the squeezed ansatz introduces nonlinear corrections to both subsystems, offering a more expressive description of coupled electron–photon dynamics.

## 4 QEDFT

Density functional theory (DFT) has become the workhorse of *ab initio* quantum chemical simulations,^86^ owing to its favorable computational scaling and its rigorous foundation in the one-to-one mapping between the real-space electronic density ρ­(**r**), the external potential *v*_ext_(**r**), and the total electronic energy.^87^ In the Kohn–Sham (KS) formulation of DFT,^88^ decades of development of increasingly accurate density functional approximations (DFAs) have extended the reach of DFT to a wide range of molecular and materials phenomena.^89^

Motivated by the success of DFT in electronic structure theory, quantum electrodynamical density functional theory (QEDFT)^48,53,70,71^ was developed as a formal extension that rigorously incorporates the quantized electromagnetic field into the DFT framework. QEDFT constructs a generalized mapping between the external scalar potential and current, and the coupled electron–photon ground-state wave function. We emphasize that QEDFT was originally formulated as a more general framework to tackle static and time-depdendent problems.^53^ By invoking different constructions for the auxiliary systems, QEDFT can be applied to the fully relativistic case with a continuum of photonic modes, as well as to the nonrelativistic limit with a few quantized cavity modes. While each limit allows us to investigate different, but equally interesting, physical phenomena, our interest in this perspective is to discuss aspects of the latter case and its applications and consequences to molecular systems.

In this section, we outline the theoretical foundations of QEDFT applied to the ground state of the Pauli–Fierz Hamiltonian,^90,91^ emphasizing how the mapping is extended to include photonic variables. We then summarize recent developments in electron–photon density functional approximations and their implementation in practical QEDFT calculations.

### 4.1 Ground State QEDFT for the Pauli–Fierz Hamiltonian in Length Gauge

As noted earlier, the success of KS-DFT relies on the existence of a one-to-one mapping between the electronic density, the external potential, and the total energy of the system. The KS formalism further introduces an auxiliary system of noninteracting particles that, subject to an effective potential, reproduces the exact interacting electronic density of the real system. A similar construction can be extended to QEDFT to treat coupled electron–photon systems.

For such systems, the Pauli–Fierz Hamiltonian in the length gauge takes the form:^53,70^

26 $$H=\sum_{i}-\frac{1}{2}\nabla_{i}^{2}+v_{\mathrm{ext}}\left(r_{i}\right)+\sum_{i<j}\frac{1}{\left|r_{i}-r_{j}\right|}+\sum_{\alpha }\left[\frac{p_{\alpha }^{2}}{2}+\frac{\omega_{\alpha }^{2}}{2}{\left(q_{\alpha }-\frac{d_{\alpha }}{\omega_{\alpha }}\right)}^{2}+\frac{j_{\mathrm{ext}}^{\alpha }}{\omega_{\alpha }}q_{\alpha }\right]$$

where *d*_α_ = **λ**_**α**_·**μ** as in QED-HF, **μ** is the molecular dipole moment, *q*_α_ and *p*_α_ are the photonic displacement and momentum operators for mode α, and **λ**_α_ defines the coupling strength of the system to that mode.

QEDFT is formally established through the proof of a one-to-one correspondence between external variables, namely, the external scalar potential *v*_ext_(**r**) and the external current *j*_ext_^α^—and internal variables: the electronic density ρ­(**r**) and the expectation value of the photonic displacement operator $\left\langle q_{\alpha }\right\rangle =\left\langle \frac{a_{\alpha }^{†}+a_{\alpha }}{\sqrt{2\omega_{\alpha }}}\right\rangle$ .^53,70^ This mapping implies that all observables of the system can, in principle, be expressed as functionals of these internal variables.

This foundational result enables the construction of a KS-like noninteracting reference system composed of electrons and photons:

27 $$\left|\Phi_{s}\right\rangle =\left|\Phi_{s}^{e}\right\rangle ⊗\left|n_{s}\right\rangle$$

where |Φ_*s*_^*e*^⟩ is a Slater determinant of KS orbitals ϕ_*i*_(**r**), and |*n*_*s*_⟩ is a photonic Fock state. These auxiliary states are defined such that they reproduce both the exact electronic density and photon displacement of the fully interacting system.

The KS orbitals are determined by solving the single-particle Schrödinger-like equation:

28 $$h_{s}\phi_{i}\left(r\right)=\left[-\frac{1}{2}\nabla^{2}+v_{s}\left(r\right)\right]\phi_{i}\left(r\right)=\varepsilon_{i}\phi_{i}\left(r\right)$$

where the effective potential *v*_*s*_(**r**) contains both electronic and electron–photon exchange–correlation terms:

29 $$v_{s}\left(r\right)=v_{\mathrm{ext}}\left(r\right)+v_{\mathrm{xc}}^{e-e}\left(r\right)+\sum_{\alpha }v_{\mathrm{xc}}^{\alpha ,e-\mathrm{ph}}\left(r\right)$$

Here, *v*_*xc*_^e–e^(**r**) captures the electronic exchange and correlation effects, while *v*_*xc*_^α,e–ph^(**r**) accounts for mode-specific electron–photon correlations. This formal structure provides the foundation for developing approximate exchange–correlation functionals that can capture light–matter hybridization effects at the density level.

### 4.2 Overview of Electron-Photon Functional Approximations

Despite the existence of formal constraints that must be satisfied by the exchange–correlation potentials,^70,92^ their exact forms remain unknown. As a result, the practical utility of QEDFT for light–matter coupled systems hinges on the development of accurate and efficient approximations.

From eq 29, it is evident that while one can leverage the broad landscape of existing density functional approximations for the electronic exchange–correlation term *v*_*xc*_^e–e^(**r**), the construction of reliable approximations for the electron–photon exchange–correlation potential *v*_*xc*_^α,e–ph^(**r**) is equally essential. Understanding the structure and origin of these mode-induced correlation terms is central to building practically useful QEDFT functionals.

It is important to emphasize that *v*_*xc*_^α,e–ph^(**r**) can be further decomposed into contributions from the bilinear light–matter coupling term, which involves the photonic displacement *q*_α_ and the total dipole moment of the system **R**, and the dipole self-energy (DSE) term, given by $\frac{1}{2}{\left(\lambda_{\alpha }\cdot R\right)}^{2}$ , which acts solely on the electronic degrees of freedom.

#### 4.2.1 Uncorrelated Electrons and Photons in QEDFT

Since its inception, several approximations to the electron–photon exchange–correlation potential *v*_*xc*_^α,e–ph^ have been proposed within the QEDFT framework. Below, we highlight, mostly in chronological order, a few strategies that have demonstrated practical success in modeling coupled electron–photon systems.

One intuitive approach is to treat the bilinear light–matter coupling at the mean-field level and to evaluate the dipole self-energy (DSE) term using an uncorrelated Hartree–Fock-like treatment. Electronic correlation is then incorporated using conventional exchange–correlation functionals developed for standard electronic DFT.^70,93^

In this approximation, the effective one-particle Kohn–Sham Fock operator *F*, expressed in an atomic orbital basis {|μ⟩}, takes the form:^47,93^

30 $$F_{\mu \nu }=T_{\mu \nu }+V_{\mu \nu }^{\mathrm{ext}}+J_{\mu \nu }+V_{\mu \nu }^{e-e,\mathrm{xc}}+O_{\mu \nu }^{\mathrm{DSE}}+J_{\mu \nu }^{\mathrm{DSE}}-K_{\mu \nu }^{\mathrm{DSE}}$$

where *T*_*μν*_ and *V*_*μν*_^ext^ are the kinetic and external potential integrals, *J*_*μν*_ is the classical Coulomb (Hartree) term, and *V*_*μν*_^e–e,xc^ denotes the electronic exchange–correlation contribution from a standard DFA. The terms *O* ^DSE^, *J* ^DSE^, and *K* ^DSE^ arise from the dipole self-energy evaluated at the Hartree–Fock level and represent one-electron, Coulomb-like, and exchange-like corrections, respectively (explicit expressions can be found in ref 93 and are analogous to the expressions in eq 13).

This formulation, commonly referred to as QED-DFT, can be traced back to the original theoretical framework of Tokatly.^70^ More recently, it has been employed to study thermochemical quantities, such as reaction energies and activation barriers, for molecules embedded in optical cavities, in the context of polaritonic chemistry.^93,94^

#### 4.2.2 Optimized Effective Potentials for QEDFT

Moving beyond the uncorrelated electron–photon limit, Pellegrini and co-workers proposed an approach based on optimized effective potentials (OEP) to capture exchange effects arising from light–matter interactions.^95–97^ In this QED-OEP formalism, the electron–photon exchange energy is expanded to include terms up to second order in the coupling strength λ_α_. The resulting expression is given by

31 $$E_{x}^{\alpha }=\sum_{i}^{\mathrm{occ}}\sqrt{\frac{\omega_{\alpha }}{8}}\left\langle \Phi_{i,\alpha }^{1}\right|\lambda_{\alpha }\cdot r\left|\phi_{i}\right\rangle +\frac{1}{4}\left\langle \Phi_{i,\alpha }^{2}\right|\lambda_{\alpha }\cdot r\left|\phi_{i}\right\rangle +c.c.$$

where the sum runs over occupied Kohn–Sham orbitals ϕ_*i*_, and “c.c.” denotes the complex conjugate of the preceding terms. The quantities |Φ_*i*,α_^1^⟩ and |Φ_*i*,α_^2^⟩ correspond to first- and second-order orbital response functions arising from the electron–photon interaction.

These response orbitals can be expressed as sums over unoccupied KS orbitals, analogous to orbital-dependent functionals encountered in RPA-type or double-hybrid DFT methods. Specifically,

32 $$\Phi_{i,\alpha }^{1}\left(r\right)=\sqrt{\frac{\omega_{\alpha }}{2}}\sum_{a}^{\mathrm{virt}}\frac{d_{\mathrm{ai}}}{\varepsilon_{i}-\varepsilon_{a}-\omega_{\alpha }}\phi_{a}\left(r\right)$$

and

33 $$\Phi_{i,\alpha }^{2}\left(r\right)=\sum_{a}^{\mathrm{virt}}d_{\mathrm{ai}}\phi_{a}\left(r\right)$$

where *d*_*ai*_ = ⟨ϕ_*a*_|**λ**_α_·**r**|ϕ_*i*_⟩ are the virtual-occupied elements of the field-weighted dipole integrals represented in the molecular orbital basis.

The corresponding QED-OEP exchange potential is obtained by taking the functional derivative of the exchange energy in eq 31 with respect to the electronic density. This yields an effective Kohn–Sham exchange potential that captures electron–photon exchange at the orbital-dependent level. Flick and co-workers later reformulated the QED-OEP framework to eliminate the dependence on unoccupied orbitals, rendering the method more computationally tractable for simulations of atoms and molecules in optical cavities.^96^

#### 4.2.3 Gradient Approximation in QEDFT

Despite its historical significance as the first QEDFT functional for *ab initio* calculations,^54,98^ the orbital-dependent nature of the QED-OEP method and its related approximation, QED-KLI,^96^ renders these approaches computationally demanding for larger systems of chemical relevance.

To address this limitation, Flick and co-workers employed an extension of the adiabatic connection and the fluctuation–dissipation theorem to formulate a more tractable electron–photon density functional.^99^ Retaining terms up to second order in the light–matter coupling constant λ_α_ (sufficient to describe single-photon processes), the electron–photon exchange energy can be expressed in terms of the electronic dynamic polarizability **α**(*iω*):

34 $$E_{x}=\frac{1}{2\pi }\sum_{\alpha }\int_{0}^{\infty }d\omega \frac{\omega^{2}}{\omega^{2}+\omega_{\alpha }^{2}}\lambda_{\alpha }\cdot \alpha \left(i\omega \right)\cdot \lambda_{\alpha }$$

To convert this into a proper density functional approximation, Flick adopted the Vydrov–Van Voorhis parametrization of the dynamic polarizability for isotropic systems.^100^ The resulting gradient approximation (GA) for the electron–photon exchange energy is given by

35 $$E_{x}^{\mathrm{GA}}\left[\rho ,\nabla \rho \right]=\frac{1}{16\pi }\sum_{\alpha }|\lambda_{\alpha }{|}^{2}\int dr\frac{\omega_{p}^{2}\left(r\right)}{\sqrt{\frac{\omega_{p}^{2}\left(r\right)}{3}+\omega_{g}^{2}\left(r\right)}+\omega_{\alpha }}$$

where ω_*p*_^2^(**r**) = 4π*ρ*(**r**) is the local plasmon frequency and $\omega_{g}^{2}\left(r\right)=C{\left|\frac{\nabla \rho \left(r\right)}{\rho \left(r\right)}\right|}^{4}$ is the effective gap frequency, with *C* = 0.0089. This GA depends solely on the density and its spatial gradient, and therefore belongs to the second rung of the metaphorical Jacob’s ladder of density functional approximations.^101^

Other local gap-based functionals have been explored in the context of correlation energies^102–104^ and kinetic-energy functionals.^105^ Fabiano and co-workers^104^ proposed the GAPc functional for correlation, based on earlier work by Krieger, Chen, Iafrate, and Savin:^102,103^

36 $$e_{g\mathrm{GAPc}}\left[\rho ,\nabla \rho \right]=\frac{H\left(r_{s},\zeta ,t^{2}\right)}{\epsilon_{c}^{\prime }\left(r_{s}\right)}$$

where *r*_*s*_ = (3/4*πρ*)^1/3^ is the local Wigner–Seitz radius, ζ = (*n*_↑_ – *n*_↓_)/*n* is the spin polarization, *t* = |∇ρ|/(2ϕ*k*_*s*_ρ) is a dimensionless gradient, $\phi =\frac{1}{2}\left[{\left(1+\zeta \right)}^{2/3}+{\left(1-\zeta \right)}^{2/3}\right]$ is a spin-scaling factor, and $k_{s}=\sqrt{4k_{F}/\pi }$ is the Thomas screening length. The denominator ϵ_*c*_^′^(*r*_*s*_) denotes the derivative of the correlation energy with respect to the gap, and *H* captures the gradient-dependent contributions.

Building on this idea, Mejia-Rodriguez and co-workers^106^ recently introduced a modified version of *e*_*g*GAPc_ to construct a new polarizability-based functional. They introduced a kinetic-energy-density-dependent switching function that interpolates between the original *e*_*g*GAPc_ and a modified form *e*_*g*GAPc2_, and used this expression within eq 35 to define a meta-GGA-level QEDFT exchange–correlation functional. This formulation effectively advances QEDFT up to the third rung of Jacob’s ladder.

#### 4.2.4 Photon Many-Body Dispersion Functional

While computationally more efficient than QED-OEP, the gradient approximation (GA) functional suffers from several limitations. A particularly significant limitation is its inability to capture anisotropic effects arising from the directional nature of photonic modes, evident in the explicit dependence of eq 35 on |**λ**_α_|^2^. Additionally, it lacks a proper treatment of higher-order photonic processes that contribute to electron–photon correlation energy.

The adiabatic connection and the fluctuation–dissipation theorem provide a natural path toward incorporating such effects. These frameworks also establish a formal connection between electron–photon exchange–correlation functionals in QEDFT and dispersion functionals in electronic DFT.^107^ Tasci and co-workers demonstrated that in both settings, the correlation energy within the random-phase approximation (RPA) can be expressed as

37 $$E_{c}^{\mathrm{RPA}}=-\frac{1}{2\pi }\int_{0}^{\infty }d\omega \sum_{n=2}^{\infty }\frac{1}{n}\mathrm{Tr}\left[{\left(G\left(i\omega \right)\chi_{0}\left(i\omega \right)\right)}^{n}\right]$$

where χ_0_ is the noninteracting electronic response function, and G is the electron–photon propagator in QEDFT (or the purely electronic propagator in conventional DFT).^108^

This formal similarity between dispersion and electron–photon correlation energies inspired the development of the photon many-body dispersion (pMBD) model,^108^ an extension of the many-body dispersion framework introduced by Tkatchenko and co-workers^109,110^ to account for long-range electron correlation within the QED context.

In pMBD, the electronic system is coarse-grained into a collection of quantum harmonic oscillators, each representing localized electronic dipole fluctuations. These oscillators interact with the quantized electromagnetic field, allowing for a seamless incorporation of photon modes. The oscillator parameters, frequencies and polarizabilities, are derived from a Hirshfeld partitioning^111^ of the molecular electron density.

This yields the pMBD Hamiltonian, schematically illustrated in Figure 3, which couples electronic and photonic degrees of freedom. The corresponding pMBD exchange–correlation energy is given by

38 $$E_{\mathrm{xc}}^{\mathrm{pMBD}}=\frac{1}{2}\sum_{k=1}^{3N+N_{p}}\Omega_{k}-\frac{1}{2}\sum_{i=1}^{N}\sum_{a=1}^{3}\omega_{\mathrm{ia}}-\frac{1}{2}\sum_{\alpha =1}^{N_{p}}\omega_{\alpha }$$

where ω_*ia*_ is the frequency of the *i* ^th^ atomic oscillator in the *a* ^th^ Cartesian direction, ω_α_ is the frequency of photonic mode α, and Ω_*k*_ denotes the eigenfrequencies of the coupled pMBD Hamiltonian.

**Figure 3:**
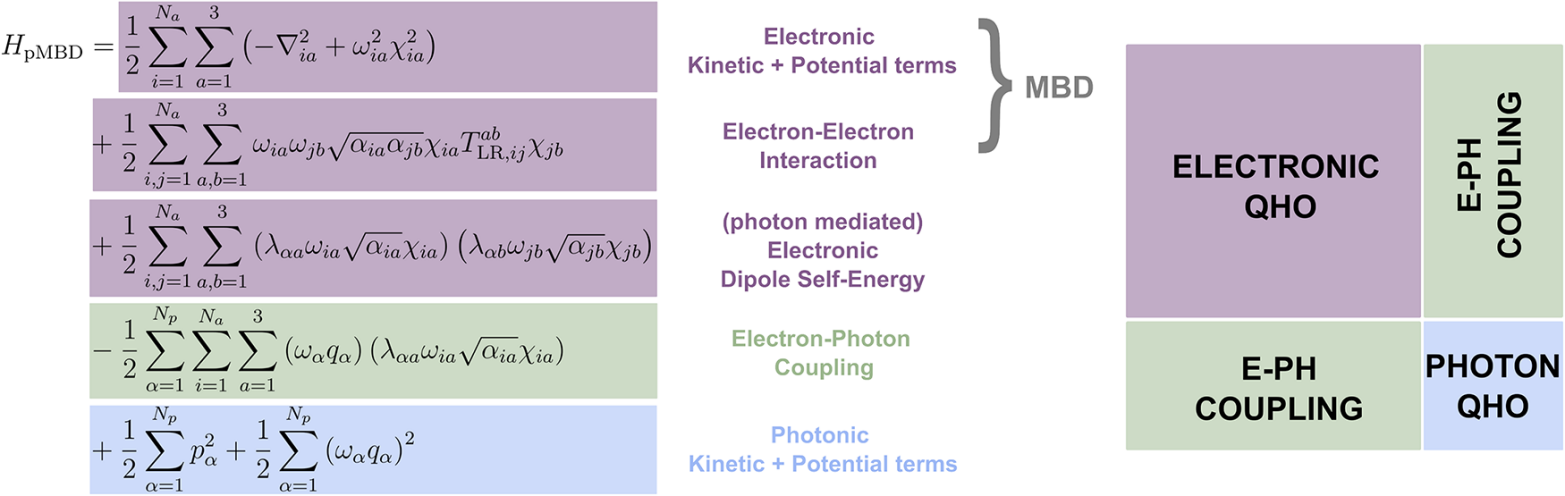
Structure of the pMBD Hamiltonian and its relation to the electronic MBD method for dispersion interactions. Reproduced from ref 108.

#### 4.2.5 Quantum Monte Carlo-Based Electron-Photon Correlation Functionals

We should also highlight recent efforts to develop QEDFT functionals by fitting accurate *ab initio* data generated from QED extensions of quantum Monte Carlo, such as QED Auxiliary Field Quantum Monte Carlo (QED-AFQMC),^112^ applied to canonical model systems like the homogeneous electron gas (HEG). This strategy mirrors the historical trajectory of electronic DFT, whose widespread adoption in condensed matter physics, materials science, and quantum chemistry was accelerated by parametrizations of the local density approximation (LDA) correlation energy,^113,114^ based on the seminal quantum Monte Carlo simulations of the uniform electron gas by Ceperley and Alder.^115^

In this context, Weber and co-workers recently developed a light–matter correlation QEDFT functional by performing QED-AFQMC simulations on a two-dimensional electron gas (2DEG) confined in a cosine-like external potential, in the absence of Coulomb electron–electron interactions.^116^

Starting from the velocity-gauge Pauli–Fierz Hamiltonian (eq 1), the authors applied a canonical transformation that eliminates the diamagnetic *A* ^2^ term. This transformation yields dressed photon modes and renormalized cavity frequencies,

39 $$\mathrm{\omega ̃}=\omega \sqrt{1+\frac{N\lambda^{2}}{V\omega^{2}}}$$

and renormalized light–matter couplings,

40 $$\mathrm{\lambda ̃}=\frac{N\lambda^{2}}{V\mathrm{\omega ̃}}$$

where *N* is the number of electrons and *V* is the cavity mode volume. These renormalized parameters allowed the authors to systematically explore the parameter space and fit a correlation functional of the form:

41 $$E_{c,\mathrm{e‐p}}=-\frac{Q^{2}}{c_{1}+\frac{c_{2}\rho^{2/D}}{\mathrm{\lambda ̃}}}$$

where *c*_1_ = 4.672 and *c*_2_ = 63.73 are constants fitted to QED-AFQMC data, and $Q^{2}$ captures the averaged gradient fluctuations of the electronic density in the polarization direction.

The quantity $Q^{2}$ can itself be expressed as a function of the dimensionless variable *x*_*v*_ = ρ^–2/*D*^ *v*, where *v* is the strength of the modulating external potential. The fitted form is

42 $$Q^{2}=\rho^{2/D}\cdot \frac{x_{v}}{b_{1}+b_{2}x_{v}+b_{3}x_{v}^{3/2}}$$

with fit parameters *b*_1_ = 3.27, *b*_2_ = −0.242, and *b*_3_ = 0.213.

This approach highlights a promising direction in QEDFT functional development, combining many-body QED simulations with density-based interpolation strategies to generate transferable, low-cost approximations that capture correlation effects in light–matter hybrid systems.

#### 4.2.6 Photon-Free Functional

We conclude this section by presenting an alternative strategy to derive electron–photon exchange–correlation approximations based on the local force-balance equation.^117,118^ This local force can be obtained by considering the equations of motion of the paramagnetic current density (***j* ^**_*p*_) and, in the context of QEDFT, is the central quantity for the derivation of the photon-free functional.^119–121^

The full derivation of the photon-free functional is out of the scope of this perspective, and we refer the interested reader to refs 120 and 121 for further details. Here, we only highlight a few important steps. Different from the other QEDFT functionals discussed so far, the derivation of the photon-free functionals starts by considering the quantized minimal coupling, Coulomb gauge Hamiltonian in eq 1 (under the long-wavelength approximation), and redefining the photonic modes (with frequency ω̃_α_ and coupling strength λ̃_α_ as defined in eqs 39 and 40, respectively) in such a way that the new Hamiltonian does not have a dependency on the diamagnetic term *Â* ^2^.^121^ In this context, we have (after dropping the zero-point energy)

43 $$Ĥ=\sum_{i}^{N_{e}}\frac{1}{2}{\mathrm{p̂}}_{i}^{2}+\mathrm{V̂}+\frac{1}{c}\hat{Ã}\cdot {Ĵ}_{p}+\sum_{\alpha }^{N_{p}}{\mathrm{\omega ̃}}_{\alpha }{ã}^{†}ã$$

with *Ĵ*_*p*_ being the paramagnetic current operator.

The local force-balance equation allows us to write the electron–photon exchange–correlation potential *v*_*pxc*_(***r***) as a Poisson-like equation

44 $$\nabla^{2}v_{\mathrm{pxc}}\left(r\right)=\frac{1}{c}\nabla \cdot \left[\frac{\left\langle \left(\hat{Ã}\cdot \nabla \right){ĵ}_{p}\left(r\right)\right\rangle }{\rho \left(r\right)}\right]$$

where ρ­(*r*) is the electronic density. A Breit-type approximation allows us to connect fluctuations of the vector potential operator (Δ*Ã̂*_α_) for each photonic mode with fluctuations in the paramagnetic density operator $\left(\Delta {Ĵ}_{p}\right)$

45 $$\Delta {\hat{Ã}}_{\alpha }\approx -c\frac{{\mathrm{\lambda ̃}}_{\alpha }^{2}}{{\mathrm{\omega ̃}}_{\alpha }^{2}}{\mathrm{\epsilon ̃}}_{\alpha }\cdot \Delta {Ĵ}_{p}$$

and, therefore, obtain an estimate for the expectation value in eq 44. Focusing only on the exchange component of *v*_*pxc*_ and assuming the homogeneous density limit, we obtain a local-density approximation (LDA) to the electron–photon exchange potential

46 $$\nabla^{2}v_{\mathrm{pxLDA}}=-\sum_{\alpha }^{N_{p}}\frac{2\pi^{2}{\mathrm{\lambda ̃}}_{\alpha }^{2}}{{\mathrm{\omega ̃}}_{\alpha }^{2}}\left[{\left({\mathrm{\epsilon ̃}}_{\alpha }\cdot \nabla \right)}^{2}{\left(\frac{\rho \left(r\right)}{2V_{d}}\right)}^{2/d}\right]$$

where *V*_d_ is the volume of the d-dimensional unit sphere. Finally, the associated electron–photon LDA exchange energy can be obtained as

47 $$E_{\mathrm{pxLDA}}\left[\rho \left(r\right)\right]=\frac{-2\pi^{2}}{(d+2){\left(2V_{d}\right)}^{2/d}}\sum_{\alpha }^{N_{p}}\frac{{\mathrm{\lambda ̃}}_{\alpha }^{2}}{{\mathrm{\omega ̃}}_{\alpha }^{2}}\int dr\rho^{2+d/d}\left(r\right)$$

## 5 QED-TDDFT

The original formulation of QEDFT was, in fact, time-dependent, establishing a rigorous foundation for modeling coupled electron and photon systems in the presence of time-dependent external perturbations.^53,70^ Linear response QEDFT^122,123^ has emerged as a practical tool for computing excitation energies, oscillator strengths, and absorption spectra of molecules in optical cavities. Recently, it was also used to investigate the connection between collective coupling, disorder, and strong local response in the electronic strong-coupling regime,^34,43,124^ and it was extended to incorporate nuclear motion and capture vibronic signatures of cavity-modified excitations.^125^

Building on this linear response framework and on the QED-DFT approximation discussed in Section 4.2.1, several groups have used time-dependent QED-DFT (QED-TDDFT) to investigate electronic strong coupling in molecular systems.^56,57,93,126^ To clarify how different approximations influence the QED-TDDFT formalism, Yang and co-workers introduced the QED-TDDFT prism,^56^ a conceptual framework that classifies QED-TDDFT methods along three dimensions. The first distinguishes whether the electronic ground state is computed in the presence of the cavity field (a relaxed reference, typically using QED-DFT or QED-HF) or is obtained from standard DFT (an unrelaxed reference). The second concerns how electron–photon exchange and correlation effects are treated. In many cases, these are neglected or handled at the mean-field level, though more sophisticated variants include orbital-dependent corrections using optimized effective potentials. The third axis considers the inclusion of dipole self-energy terms in the response equations, which may be omitted, partially included, or fully included depending on the formulation. Together, these choices define a spectrum of methods, from efficient unrelaxed mean-field approaches to fully correlated relaxed variants. The prism provides a systematic view of how methodological differences manifest in computed spectra and polaritonic states.

In typical QED-TDDFT implementations, excited states are expressed as

48 $$\left|\Psi_{I}\right\rangle ={Ô}_{I}^{†}\left|\Phi_{0}\right\rangle$$

with the excitation operator defined as

49 $${Ô}_{I}^{†}=\sum_{\mathrm{ia}}\left(X_{\mathrm{ia}}^{I}{â}_{a}^{†}{â}_{i}-Y_{\mathrm{ia}}^{I}{â}_{i}^{†}{â}_{a}\right)+M^{I}{\mathrm{b̂}}^{†}-N^{I}\mathrm{b̂}$$

Following Rowe’s equation-of-motion formalism,^127^ this ansatz leads to a generalized eigenvalue equation of the form

50 $$\left(\begin{array}{llll} A+\Delta & B+\Delta^{\prime } & g^{†} & g^{†}\\ B+\Delta^{\prime } & A+\Delta & g^{†} & g^{†}\\ g & g & \omega_{\mathrm{cav}} & 0\\ g & g & 0 & \omega_{\mathrm{cav}} \end{array}\right)\left(\begin{array}{l} X\\ Y\\ M\\ N \end{array}\right)=\Omega \left(\begin{array}{llll} 1 & 0 & 0 & 0\\ 0 & -1 & 0 & 0\\ 0 & 0 & 1 & 0\\ 0 & 0 & 0 & -1 \end{array}\right)\left(\begin{array}{l} X\\ Y\\ M\\ N \end{array}\right)$$

Here, ***A*** and ***B*** are the conventional TDDFT matrices obtained from the electronic Hamiltonian. The matrices **Δ** and **Δ**′ represent dipole self-energy contributions, defined as

51 $$\Delta_{\mathrm{ai},\mathrm{bj}}=d_{\mathrm{ai}}d_{\mathrm{jb}}-d_{\mathrm{ab}}d_{\mathrm{ij}}$$

52 $$\Delta_{\mathrm{ai},\mathrm{bj}}^{\prime }=d_{\mathrm{ai}}d_{\mathrm{bj}}-d_{\mathrm{aj}}d_{\mathrm{ib}}$$

where the dressed dipole integrals are given by

53 $$d_{\mathrm{ai}}=-\sum_{\xi \in \left\{x,y,z\right\}}\lambda_{\xi }\int \phi_{a}^{*}\left(r\right)r_{\xi }\phi_{i}\left(r\right)d\tau$$

The choice of electronic Hamiltonian in the double commutator, whether it is the Pauli–Fierz Hamiltonian *Ĥ*_PF_ or the coherent-state transformed Hamiltonian *Ĥ*_CS_, determines whether the formulation is unrelaxed or relaxed, respectively. The unrelaxed formalism of ref 56 is recovered by using *Ĥ*_PF_ and omitting exchange terms in **Δ** and **Δ**′.

Systems explored by Yang and co-workers include formaldehyde, benzaldehyde, pyrrole, and coumarin derivatives.^56,57^ These studies revealed how different regions of the QED-TDDFT prism affect Rabi splitting magnitudes, state reordering, and cavity detuning sensitivity. Further work extended QED-TDDFT to small unsaturated hydrocarbons and carbonyl-containing molecules,^93,122^ as well as to larger sets of photoactive systems for benchmarking exchange–correlation functionals.^126^ These applications highlight the versatility and scope of QED-TDDFT for describing light–matter hybridization and cavity-modified excited-state dynamics.

## 6 QED-CI

A general QED configuration interaction ansatz for the Pauli–Fierz (or other *ab initio* QED Hamiltonians) can be written as follows,

54 $$\left|\Psi \right\rangle =\sum_{n}\sum_{I}C_{I,n}\left|\Phi_{I}^{e}\right\rangle ⊗\left|n^{p}\right\rangle$$

where the key difference between the QED-CI ansatz and a CI ansatz for the electronic Hamiltonian is that each term in QED-CI expansion is a tensor product between a many-electron state, |Φ_*I*_^e^⟩, and a photonic state, |*n* ^p^⟩. Formulations of QED-CIS,^47,58^ QED-CASCI,^59^ and QED-FCI^59,82^ have used Slater determinants as the many-electron basis, and either photon occupation states or coherent states as the photonic basis. In principle, other many-electron basis states (e.g., configuration state functions), and other photonic basis functions, are possible as well. While the CIS method is polynomial scaling, FCI and CASCI methods have factorial scaling with the number of electrons and orbitals that comprise the excitation space. The construction and diagonalization of the full Hamiltonian matrix for active spaces larger than 10 or so electrons becomes infeasible for most computer hardware. The iterative method to avoid explicit building and storage of the Hamitonian CI matrix (direct CI) for configuration state function (CSF) and determinant basis was invented in the 1980s; leveraging further algorithmic and hardware advances, this approach has been successfully applied to systems up of 22 electrons in 22 orbitals.^128,129^ Thus, practical implementations of QED-CASCI and QED-FCI will rely on generalizing the direct CI algorithm to iteratively solve for the desired roots of coupled wave functions in the tensor product space of electronic and photonic configurations.

Here, we briefly present the direct CI method in determinant basis extended for the CS-PF Hamiltonian and CAS wave function. First, for the CI wave function described by eq 54 where determinants can only belong to an active space, the PF Hamiltonian can be equivalently represented by the CAS PF Hamiltonian:

55 $${Ĥ}_{\mathrm{PF}}^{\mathrm{CAS}}=E_{c}+\frac{1}{2}d_{N}^{2}+\omega {\mathrm{b̂}}^{†}\mathrm{b̂}+\sum_{\mathrm{tu}}F_{\mathrm{tu}}^{c}{Ê}_{\mathrm{tu}}+\frac{1}{2}\sum_{\mathrm{tuvw}}{\left(\mathrm{tu}|\mathrm{vw}\right)}^{\prime }{Ê}_{\mathrm{tu},\mathrm{vw}}-\sqrt{\frac{\omega }{2}}\sum_{\mathrm{tu}}d_{\mathrm{tu}}{Ê}_{\mathrm{tu}}\left({\mathrm{b̂}}^{†}+\mathrm{b̂}\right)-\sqrt{\frac{\omega }{2}}\left(d_{N}+2\sum_{i}d_{\mathrm{ii}}\right)\left({\mathrm{b̂}}^{†}+\mathrm{b̂}\right)$$

and the CS-PF Hamiltonian can be substituted by the CAS CS-PF Hamiltonian:

56 $${Ĥ}_{\mathrm{CS}-\mathrm{PF}}^{\mathrm{CAS}}=E_{c}+\frac{1}{2}\langle d_{e}\rangle^{2}+\omega {\mathrm{b̂}}^{†}\mathrm{b̂}+\sum_{\mathrm{tu}}F_{\mathrm{tu}}^{c}{Ê}_{\mathrm{tu}}+\frac{1}{2}\sum_{\mathrm{tuvw}}{\left(\mathrm{tu}|\mathrm{vw}\right)}^{\prime }{Ê}_{\mathrm{tu},\mathrm{vw}}-\sqrt{\frac{\omega }{2}}\sum_{\mathrm{tu}}d_{\mathrm{tu}}{Ê}_{\mathrm{tu}}\left({\mathrm{b̂}}^{†}+\mathrm{b̂}\right)+\sqrt{\frac{\omega }{2}}\left(\left\langle d_{e}\right\rangle -2\sum_{i}d_{\mathrm{ii}}\right)\left({\mathrm{b̂}}^{†}+\mathrm{b̂}\right)$$

Here, the operators *Ê*_*tu*_ and *Ê*_*tuvw*_ are given by

57 $${Ê}_{\mathrm{tu}}=\sum_{\sigma =\alpha ,\beta }a_{t\sigma }^{†}a_{u\sigma }$$

58 $${Ê}_{\mathrm{tu},\mathrm{vw}}={Ê}_{\mathrm{tu}}{Ê}_{\mathrm{vw}}-\delta_{\mathrm{uv}}{Ê}_{\mathrm{tw}}$$

We define the modified one- and two-electron contributions *h*_*pq*_^′^ and (*pq*|*rs*)′, core Fock contributions *F*_*pq*_^c^, and core energy *E*_c_ as

59 $${h_{\mathrm{pq}}}^{\prime }=h_{\mathrm{pq}}-\frac{1}{2}q_{\mathrm{pq}}-\left\langle {\mathrm{d̂}}_{e}\right\rangle d_{\mathrm{pq}}$$

60 $${\left(\mathrm{pq}|\mathrm{rs}\right)}^{\prime }=\left(\mathrm{pq}|\mathrm{rs}\right)+d_{\mathrm{pq}}d_{\mathrm{rs}}$$

61 $$F_{\mathrm{pq}}^{c}={h^{\prime }}_{\mathrm{pq}}+\sum_{i}2{\left(\mathrm{pq}|\mathrm{ii}\right)}^{\prime }-{\left(\mathrm{pi}|\mathrm{qi}\right)}^{\prime }$$

62 $$E_{c}=\sum_{i}{h^{\prime }}_{\mathrm{ii}}+F_{\mathrm{ii}}^{c}$$

As was discussed in relation to the QED-HF Fock matrix (eqs 12 and 13), *d*_*pq*_ are field weighted dipole integrals and *q*_*pq*_ are field-weighted quadrupole integrals.

The matrix-vector product of the CAS CS-PF Hamiltonian is given by

63 $$\sigma_{I,m}=\sum_{\mathrm{Jn}}\left\langle \Phi_{I}^{e},m^{p}\right|{Ĥ}_{\mathrm{CS}-\mathrm{PF}}^{\mathrm{CAS}}\left|\Phi_{J}^{e},n^{p}\right\rangle C_{J,n}$$

64 $$=\sum_{\mathrm{tu}}F_{\mathrm{tu}}^{c}\left\langle \Phi_{I}^{e}\right|{Ê}_{\mathrm{tu}}\left|\Phi_{J}^{e}\right\rangle C_{J,m}+\frac{1}{2}\sum_{\mathrm{tuvw}}{\left(\mathrm{tu}|\mathrm{vw}\right)}^{\prime }\left\langle \Phi_{I}^{e}\right|\left({Ê}_{\mathrm{tu}}{Ê}_{\mathrm{vw}}-\delta_{\mathrm{uv}}{Ê}_{\mathrm{tw}}\right)\left|\Phi_{J}^{e}\right\rangle C_{J,m}-\sqrt{\frac{\omega }{2}}\sum_{\mathrm{tu}}d_{\mathrm{tu}}\left\langle \Phi_{I}^{e}\right|{Ê}_{\mathrm{tu}}\left|\Phi_{J}^{e}\right\rangle \left(\sqrt{m}C_{J,m-1}+\sqrt{m+1}C_{J,m+1}\right)+\sqrt{\frac{\omega }{2}}(\left\langle {\mathrm{d̂}}_{e}\right\rangle -2\sum_{i}d_{\mathrm{ii}})\left(\sqrt{m}C_{I,m-1}+\sqrt{m+1}C_{I,m+1}\right)+\left(E_{c}+m\omega +\frac{1}{2}\langle {\mathrm{d̂}}_{e}\rangle^{2}\right)C_{I,m}$$

To avoid the explicit construction of the Hamiltonian, the direct CI algorithm takes advantage of the sparse structure of the coupling matrix ⟨Φ_*I*_^*e*^|*Ê*_*tu*_|Φ_*J*_^*e*^⟩. Only the nonzero elements of the coupling matrix are stored, and the storage requirement is further reduced, considering that the coupling matrix can be separated into the α- and β-spin blocks. A representative calculation from the QED-CASCI approach is shown in Figure 4. In particular, we consider correlating 12 electrons in 12 orbitals with 10 photonic Fock states to resolve the polariton states that arise by coupling a cavity mode to the *S*_0_ → *S*_2_ transition (the first optically bright transition) along with a nearby triplet state (*T*_3_). This illustrates how the Rabi splitting between the polariton states can push the (singlet) lower polariton energy below the triplet energy under strong light–matter coupling. Although the triplet state is optically dark, it still experiences cavity effects through the dipole self-energy, specifically through quadrupole terms.

**Figure 4:**
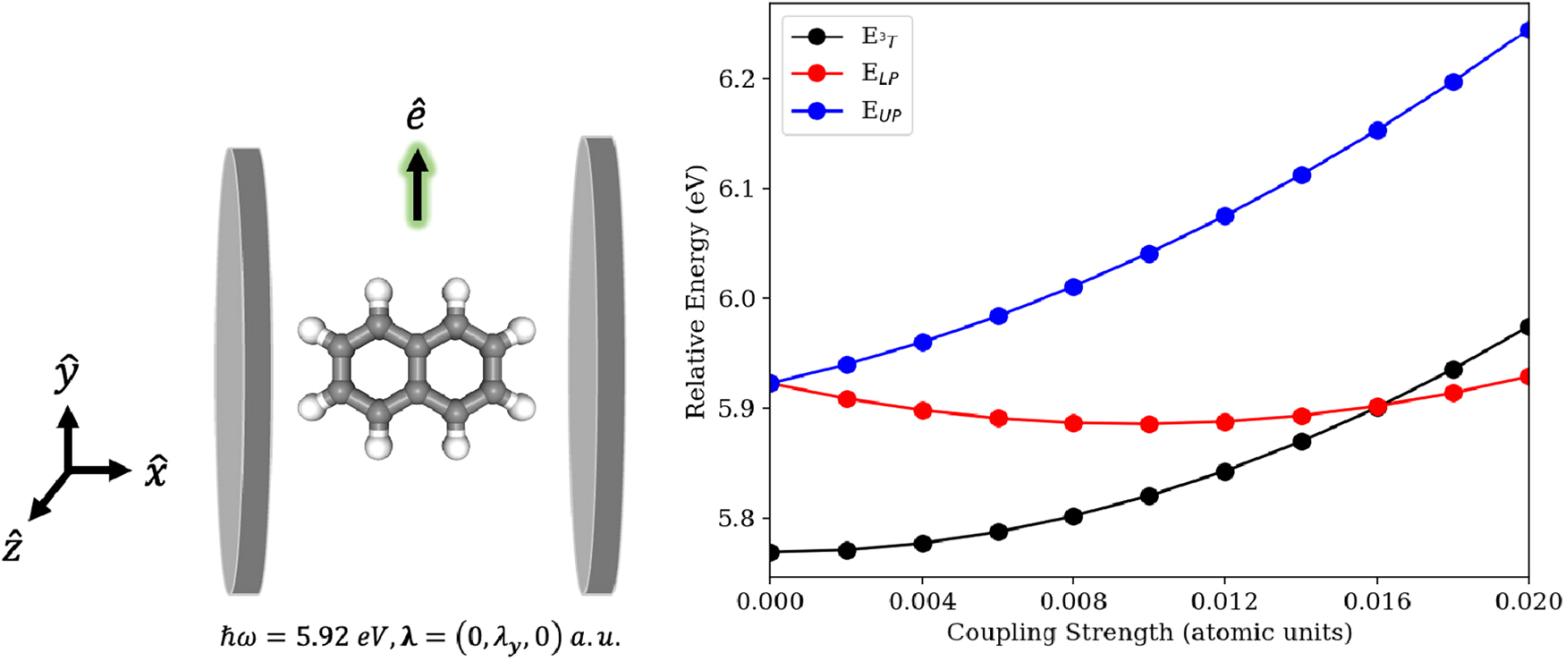
(Left) Schematic of the naphthalene coupled to a cavity mode polarized along the *y*-axis and ℏω = 5.92 eV. (Right) Relative energies of the polariton states emerging from coupling the *S*_0_ → *S*_2_ transition to the cavity mode plotted along with the relative energy of a nearby triplet state *T*_3_. The coupled energies are computed using QED-CASCI­(12e,12o,10ph)/cc-pVTZ and are plotted relative to the ground state from the CASCI­(12e,12o)/cc-pVTZ level. Reproduced from ref 59. under permission of the CC-BY 4.0 License.

## 7 QED-CASSCF

In the QED-CASCI and QED-FCI approaches, only the CI expansion coefficients are subject to variational optimization, while the orbital basis is kept fixed. In the limit of QED-FCI with a saturated photonic basis, the energies are invariant to orbital rotations, and so the choice of orbital basis is not crucial. However, for QED-CASCI, the selection of a suitable orbital basis is imperative for ensuring good accuracy. Similar to in traditional electronic structure theory, one possible approach is to self-consistently optimize the electronic wave function within a given active space and the orbital basis. Such approaches are referred to as multiconfigurational self-consistent field (MCSCF) or complete active space self-consistent field (CASSCF) approaches.^130–132^

In QED extensions of this approach, one may define the QED-CASSCF energy for the CAS CS-PF Hamiltonian as

65 ECS−PFCAS=EcCS+∑mmω(CI,m)2+∑tuFtucDtu+∑tuvw12(tu|vw)′Dtu,vw−ω2∑tudtu(Dpe)tu+ω2(⟨de⟩−2∑idii∑Im(mCI,mCI,m−1+Mm+1CI,mCI,m+1)

where *D*_*tu*_ and *D*_*tu*,*vw*_ are elements of the one- and two-electron reduced density matrices, respectively, and (*D*_*pe*_)_*tu*_ is an element of the electron–photon reduced density matrix. This energy expression is then variationally optimized with respect to the orbital basis, which directly impacts the integrals and Fock matrix terms, and the wave function coefficients, which directly impact the density matrix elements. This is an iterative procedure that alternates between linear variational steps for the wave function, and then a nonlinear optimization of the orbitals. One may apply this procedure to ground and excited states, and so there are multiple ways to define this energy expression. One approach is to express the energy for a single state, wherein the wave function coefficients (*C*_Im_) and density matrix elements correspond to a single desired energy eigenstate (which may be the ground state, or may be an excited state of interest). Such an approach is referred to as a state-specific approach, and recently, a state-specific QED-CASSCF was developed by Ronca and co-workers.^133^ Alternatively, one may compute these quantities from an average over two or more states, such that the energy corresponds to a weighted average over those states. This SA-QED-CASSCF approach was also recently developed by Vu and co-workers.^60^

We briefly demonstrate the SA-QED-CASSCF approach using the magnesium hydride cation as a model system. In this example, we examine coupling to a photon with an energy of 3.7 eV that facilitates the *S*_0_ → *S*_1_ transition at approximately *r* = 2.2 Å. The exact QED-FCI energy is approximated using QED-DMRG with a saturated photonic basis of 10 Fock states. For our QED-CASSCF calculation, we select an active space of 8 electrons in 10 orbitals and apply equal weighting when averaging over the ground, lower-polariton, and upper-polariton states. We also analyze QED-CASCI with a fixed RHF orbital basis within the same active space, using the cc-pVDZ basis set throughout all calculations. The results show remarkably close agreement between QED-CASSCF and QED-DMRG for all states and coupling conditions, while QED-CASCI results deviate noticeably under all conditions. This demonstrates the potential importance of simultaneous optimization of both orbitals and wave functions in QED-CAS calculations (Figure 5).

**Figure 5:**
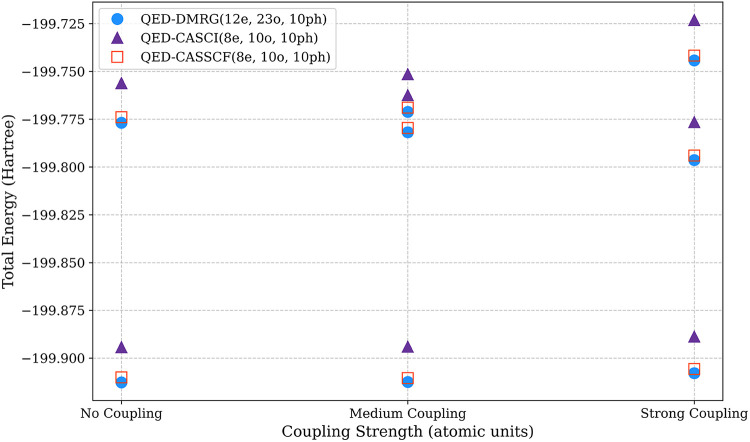
Cavity-free (No Coupling) energies of the *S*_0_ and *S*_1_ state of the magnesium hydride cation compared to the coupled ground, lower-, and upper-polariton energies at medium (λ_*z*_ = 0.01 atomic units) and strong (λ_*z*_ = 0.05 atomic units) coupling as computed by QED-CASSCF and QED-CASCI with 8 active electrons in 10 active orbitals and QED-DMRG that correlates all 12 electrons into all 23 orbitals in a cc-pVDZ basis set. We use a saturated photonic basis with 10 Fock states in each calculation.

## 8 QED-CC

A variety of QED generalizations of *ab initio* many-body methods have been developed in order to capture electron–photon correlation effects that are missing in mean-field QED approaches. The motivation for the QED-CC framework, in particular, stems from the success of standard CC expansions in electronic structure theory;^134^ this success is derived from the efficiency of the exponential *ansatz* of CC theory in capturing correlation effects. In 2020, two groups independently introduced similar QED-CC formalisms to incorporate explicit electron–photon correlations in *ab initio* molecular calculations.^49,62^ On one hand, the use of nilpotent boson operators in the “polaritonic” CC theory of ref 62 leads to a linear parametrization of the photon space, whereas, on the other hand, the use of conventional boson operators in the QED-CC approach of ref 49 results in a nonlinear parametrization one expects in CC theory. In either case, these works inspired numerous additional developments that are described below.

Following and building upon the formalism in ref 49, the QED-CC wave function is |Ψ_CC_⟩ = exp­(*T̂*)|Φ_0_⟩, with |Φ_0_⟩ = |0_e_⟩|0⟩. The cluster operator is

66 T̂=∑np=1MpT̂0,np+∑ne=1Me∑np=0MpT̂ne,np;T̂ne,np=(1ne!)2ta1...anei1...ine[np](b̂†)np∏k=1ne(âak†âik)

where sums over occupied or unoccupied spin orbitals (*i*_*k*_ and *a*_*k*_, respectively) are implied. The quantities *M*_e_ and *M*_p_ are preselected truncation levels for the electron and photon parts of the cluster operator, and ^[*n*_*p*_]^ *t*_*a*_1_...*a*_*ne*__^*i*_1_...*i*_*ne*_^ are *n*_e_-electron–*n*_p_-photon cluster amplitudes. The choice of *M*_e_ and *M*_p_ traverses the QED-CC hierarchy, with *M*_e_ = 2 and *M*_p_ = 1 giving the QED-CCSD-21 method,^49^ *M*_e_ = 2 and *M*_p_ = 2 leading to QED-CCSD-22,^135^ etc. The cluster amplitudes are obtained in the same way as in standard CC theory, by solving projective equations involving the similarity-transformed Pauli–Fierz Hamiltonian (in the coherent-state basis), *H̅* = exp­(−*T̂*) *Ĥ*_CS_ exp­(*T̂*).

Extending QED-CC to treat excited states via the equation-of-motion (EOM) framework^134,136–138^ follows the same logic. The wave function for an excited state *I* is represented by right- and left-hand states:

67 $$\left|\Psi_{I}\right\rangle ={\mathrm{R̂}}_{I}\exp\left(\mathrm{T̂}\right)\left|\Phi_{0}\right\rangle ,\mathrm{and},\left\langle {\mathrm{\Psi ̃}}_{I}\right|=\left\langle \Phi_{0}\right|{\mathrm{L̂}}_{I}\exp\left(-\mathrm{T̂}\right)$$

where *R̂*_*I*_ and *L̂*_*I*_ are linear excitation operators that are expanded in a many-electron/photon basis, as was done for the cluster amplitudes in eq 65. Excited-state wave functions and energies are then obtained by solving

68 $$\mathrm{H̅}{\mathrm{R̂}}_{I}\left|\Phi_{0}\right\rangle =E_{I}{\mathrm{R̂}}_{I}\left|\Phi_{0}\right\rangle ,,\left\langle \Phi_{0}\right|{\mathrm{L̂}}_{I}\mathrm{H̅}=E_{I}\left\langle \Phi_{0}\right|{\mathrm{L̂}}_{I}$$

where *E*_*I*_ is the energy of state *I*.

Shortly after the introduction of QED-CC, several groups adopted and extended the approach to study cavity modifications to various ground-state molecular properties. It was shown^63^ that strong-coupling to electronic degrees of freedom can lead to nontrivial modifications to electron affinities (EAs) of small molecules, while also demonstrating that QED-HF significantly overestimates cavity effects, relative to QED-CCSD-21. Unitary formulations of QED-CCSD-21 and QED-CCSD-22 were implemented for quantum computing applications,^135^ showing that ignoring two-electron-two-photon correlations can lead to notable numerical discrepancies in electron attachment energies. Topics and applications such as cavity-mediated selectivity in the Diels–Alder^139,140^ and azide–alkyne^141^ cycloaddition reactions, and whether vacuum fluctuations could catalyze/inhibit proton-transfer reactions^94^ have been explored in recent years. For the treatment of larger systems with QED-CC, a “QED-CC-in-QED-SCF” embedding protocol was proposed to localize photon–electron correlations and address scaling challenges in larger *ab initio* calculations.^142^

The QED-CCSD-21, QED-DFT, and QED-FCI approaches have been applied to inspect how vacuum fluctuations affect intermolecular forces, including van der Waals interactions.^143,144^ They found that mean-field QED approaches (QED-HF and QED-DFT) are poor descriptors for intermolecular interactions within cavity-bound systems, compared to correlated treatments (QED-CC and QED-FCI). One of the more notable observations in that work was that the dipole self-energy term alters the familiar *R* ^–6^ scaling law for dispersion-type interactions to include an *R* ^–3^ component. Another key takeaway was that cavity-mediated correlations may extend over unexpectedly large distances. Other works have applied QED-CC to the collective strong-coupling regime with multiple interacting molecules. Machine-learning-based interaction potentials derived from QED-CC simulations were applied in ref 144 to hundreds of H_2_ molecules within a cavity, revealing orientation-dependent intermolecular forces and phase behavior unique to the strong-coupling regime. A more recent attempt to reach the collective strong-coupling regime with QED-CC-based approaches can be found in ref 145. There, the single-molecule and collective strong-coupling limits for an argon excimer were studied, providing insights into mechanisms and consequences of light–matter coupling in each limit. The QED-CC method has also been generalized for the description of molecules coupled to optical cavities that support chiral modes.^146,147^

The preceding examples focused on cavity-induced changes to ground-state electronic structure. Regarding the description of excited states, the original polaritonic CC^62^ and QED-CC^49^ papers described the extension of these approaches within the EOM-CC framework. In ref 49, the QED-EOM-CCSD-21 formalism was derived and demonstrated that electronic strong-coupling can introduce gaps in conical intersections, thereby offering a route to cavity-based control over photochemical dynamics (similar observations and suggestions have been made using QEDFT methodologies^148^). In ref 149, nonparticle-conserving QED-EOM-CC expansions were developed, targeting electron-attached excited states of open-shell systems in the QED-EA-EOM-CC approach. Continuing in a similar vein, the ionization potential (IP) variant of QED-EOM-CC was formulated in ref 150. That approach included a rigorous treatment of the ionized free electron within the cavity, which was shown to have a significant impact on IPs for the molecules in that study. Numerical properties of QED-EOM-CC formulated using the bare Pauli–Fierz Hamiltonian versus its representation in the coherent-state basis have shown that QED-EOM-CC is far less sensitive to the representation of the Hamiltonian than QED-TDDFT.^126^ The QED-CC and QED-EOM-CC models have also been generalized for use in additional contexts, including the description of molecules coupled to optical cavities that support chiral modes^151^ and molecules coupled to plasmonic excitations.^152,153^

Several other technical and intellectual advances have practical implications for the application of QED-CC/QED-EOM-CC to interesting systems. For example, a distributed-memory QED-CC code within the ExaChem program has been developed,^154^ which paves the way for routine, large-scale many-body QED simulations. ExaChem utilizes the TAMM infrastructure for efficient tensor contractions, allowing scalable simulations on HPC platforms.^155^ The derivation and implementation of linear response equations^156^ and analytical energy gradients for QED-CC^157^ represent another major advance, as such capabilities will be useful in molecular dynamics simulations of cavity-bound systems, as well as for exploring cavity-induced effects on molecular structures. Similarly, diabatic approaches to QED-CC have been built to produce and study vibrational spectra of cavity-coupled systems.^158^ Along with the embedding protocols mentioned above^142^ and strategies to approach the collective strong-coupling limit,^144,145^ these advances bring QED-CC/QED-EOM-CC closer to mainstream adoption. At the same time, additional theoretical developments expand the range of systems to which these methods can be applied. For example, to address strong electron–electron correlation, a multireference QED-CC approach that treats electron–photon and electron–electron correlations on equal footing has been developed,^159^ an important step toward applying QED-CC in strongly correlated regimes.

## 9 QED-QMC

Quantum Monte Carlo (QMC) methods provide powerful tools for studying strongly correlated light–matter systems in molecular QED. In this section, we focus on three QMC approaches: (1) Diffusion QMC (DQMC), which projects the ground state in real space and is particularly effective for few-electron, many-boson systems; (2) a hybrid AF-DQMC scheme, where electronic DOFs are treated with auxiliary fields and photonic modes with diffusion dynamics, (3) fully second-quantized Auxiliary-Field QMC (AFQMC)^112^ that is extended to include electron–photon interactions via Hubbard-Stratonovich transformations, and (4) Neural network quantum state approach. We summarize the key ideas behind each method and discuss their applicability to polaritonic systems.

### 9.1 Diffusion QMC Method

In many cases, the approximate solution to a high-dimensional integral problem can be achieved using a Monte Carlo approach, which leverages the concept of random variables in a high-dimensional space. Monte Carlo integration is ubiquitous in the community for both classical and quantum mechanical problems, when the number of degrees of freedom (DOFs) is large. One of the widely used QMC methods is the diffusion quantum Monte Carlo (DQMC) method.^160–166^ In DQMC, the wave function is approximated by a basis of random walkers whose “motion” is defined by multiple applications of a short-time Green’s function for the Schrödinger equation in imaginary time.

The imaginary time Schrödinger equation, in the high-dimensional real-space basis, can be written as

69 $$\frac{\partial }{\partial \tau }\psi \left(r,\tau \right)=\left(\frac{1}{2}\nabla_{r}^{2}+V\left(r\right)\right)\psi \left(r,\tau \right)$$

where τ = *it* and $\frac{1}{2}\nabla_{r}^{2}$ is the kinetic operator for electron. Note that the nuclear positions **R** are kept fixed in solving the electronic structures, which are dropped from the notation for brevity. The formal solution of this equation can be written as a Green’s function ψ­(**r**, τ) = ∫d**r**′*G*(**r**, **r**′, τ) ψ­(**r**′, 0). This propagator *G*(**r**, **r**′, τ) can be approximated by the Trotter–Suzuki splitting of the time-evolution operator as^167,168^ $e^{\left(\frac{1}{2}\nabla_{r}^{2}+V\left(r\right)\right)d\tau }\approx e^{1/2\nabla_{r}^{2}d\tau }e^{V\left(r\right)d\tau }$ during a short-time interval $d\tau =\frac{\tau }{N_{\mathrm{steps}}}$ , leading to the following Green’s function approach,

70 $$G\left(r,r^{\prime },\tau \right)=\lim_{d\tau \to 0}{\left[G_{\mathrm{Diff}}\left(r,r^{\prime },d\tau \right)G_{\mathrm{Birth}/\mathrm{Death}}\left(r,r^{\prime },d\tau \right)\right]}^{N_{\mathrm{steps}}}$$

The formal solutions to these Green’s functions are

71 $$G_{\mathrm{Diff}}\left(r,r^{\prime },d\tau \right)=e^{-(r-r\prime {)}^{2}/2d\tau }$$

72 $$G_{\mathrm{Birth}/\mathrm{Death}}\left(r,r^{\prime },d\tau \right)=e^{-d\tau V\left(r\right)+V\left(r\prime \right)/2}$$

Here, and in the equations above, **r** and **r**′ are two system configurations of the 3*N*_el_ dimensional space (the nuclear DOFs are fixed), where *N*_el_ is the number of electrons. The first Green’s function is the solution to the diffusion equation in free space (i.e., ∂_τ_ψ = 1/2∇^2^ψ), which leads to an unbiased Gaussian random walk with a standard deviation √τ.

The second propagator gives rise to an exponential probability of the random walker itself, often called the Birth/Death algorithm.^169^ This propagator dictates the multiplication or destruction of a walker according to the probability distribution $P_{w}∼G_{\mathrm{Birth}/\mathrm{Death}}\left(r^{\prime },r,\tau \right)$ , given the current *V*(**r**′) and previous *V*(**r**) total potential energies (for a single configuration of particles) of the system with configurations **r**′ and **r**, respectively. This term gives rise to a variable number of random walkers, which can lead to an exponential increase (or decrease), of the number of walkers.

The DQMC scheme converges to the exact solution for all nodeless ground states, which encompasses up to two Fermions (e.g., electrons) and, in principle, an infinite number of Bosons (e.g., photon modes). This convergence is ensured when a sufficiently small propagation time step *dτ* is chosen and an adequate number of random walkers *N*_w_ is used. Extensions of this scheme to ground states that have nodes (or phase changes) and to excited states have been well-studied for electronic systems.^170^ This augmentation often involves the fixed-node approximation, which necessitates *a priori* knowledge of the wave function’s nodal structure. This structure is typically derived from a Hartree–Fock–Slater determinant or its post-Hartree–Fock counterparts.^171,172^

While DQMC has been proven to exactly solve the ground state of Bosonic systems, its extension toward entangled Boson–Fermion systems, particularly those arising from the strong coupling of molecular systems to light in optical or plasmonic cavities, remains under-explored. Nevertheless, extending this method to account for one or several quantized cavity or plasmonic modes, is relatively straightforward using a similar approach by decomposing the exact Green’s function into multiple short-time propagators for both the electronic and photonic DOFs.

Namely, we start from the imaginary-time Schrödinger equation for the Pauli–Fierz Hamiltonian in the position representation for both the electrons **r** and cavity mode *q*_c_,

73 $$\frac{\partial }{\partial \tau }\psi \left(r,q_{c},\tau \right)=\left(\frac{\nabla_{r}^{2}}{2}+V\left(r\right)+\frac{\nabla_{q_{c}}^{2}}{2}+V_{\mathrm{el}-\mathrm{ph}}\left(r,q_{c}\right)+V_{\mathrm{DSE}}\left(r\right)\right)\times \psi \left(r,q_{c},\tau \right)$$

where **r** again signifies all real-space coordinates of electrons.

Utilizing the Trotter expansion, the kinetic energy of the electrons and photon can be split into one short-time Green’s function propagator, while the potential terms can be split into another set as

74 $$e^{\left(\frac{\nabla_{r}^{2}}{2}+V\left(r\right)+\frac{\nabla_{q_{c}}^{2}}{2}+V_{\mathrm{el‐ph}}\left(r,q_{c}\right)+V_{\mathrm{DSE}}\left(r\right)\right)d\tau }$$

75 $$\approx e^{\left(\frac{\nabla_{r}^{2}}{2}+\frac{\nabla_{q_{c}}^{2}}{2}\right)d\tau }e^{\left(V\left(r\right)+V_{\mathrm{el‐ph}}\left(r,q_{c}\right)+V_{\mathrm{DSE}}\left(r\right)-E_{T}\right)d\tau }\approx e^{\left(\frac{\nabla_{r}^{2}}{2}+\frac{\nabla_{q_{c}}^{2}}{2}\right)d\tau }e^{\left(V_{\mathrm{total}}\left(r,q_{c}\right)-E_{T}\right)}$$

over a short-time-interval *dτ*. Here, *V*_total_(**r**, *q*_c_) = *V*(**r**) + *V*_el‑ph_(**r**, *q*_c_) + *V*_DSE_(**r**) encompasses all the potential terms. We have also integrated the trial energy *E*_T_ directly into the above expression. This leads to the following Green’s function approach for the coupled electron–photon system,

76 $$G\left(r,q_{c},r^{\prime },{q_{c}}^{\prime },\tau \right)=\lim_{d\tau \to 0}{\left[G_{\mathrm{Diff}}\left(r,q_{c},r^{\prime },{q_{c}}^{\prime },d\tau \right)\times G_{\mathrm{Birth}/\mathrm{Death}}\left(r,q_{c},r^{\prime },{q_{c}}^{\prime },d\tau \right)\right]}^{N_{\mathrm{steps}}}$$

In this case, the inclusion of the photonic DOF is formally analogous to an additional effective electronic one with a modified configurational potential energy *V*(**r**) → *V*(**r**) + *V*_el–ph_(**r**, *q*_c_) + *V*_DSE_(**r**) = *V*_Total_(**r**, *q*_c_) dependent on its position *q*_c_ and the configurational electronic dipole μ­(**r**) as well as its square μ^2^(**r**).

The formal solutions to these Green’s functions are

77 $$G_{\mathrm{Diff}}\left(r,q_{c},r^{\prime },{q_{c}}^{\prime },d\tau \right)=e^{-|r-r\prime {|}^{2}/2d\tau }e^{-(q_{c}-q_{c}\prime {)}^{2}/2d\tau }$$

78 $$G_{\mathrm{Birth}/\mathrm{Death}}\left(r,q_{c},r^{\prime },{q_{c}}^{\prime },d\tau \right)=e^{-d\tau \left(\frac{V_{\mathrm{total}}\left(r,q_{c}\right)-V_{\mathrm{total}}\left(r\prime ,q_{c}\prime \right)}{2}-E_{T}\right)}$$

Updating the trial energy *E*_T_ follows the same procedure as with photon-free propagation.^64^ The coupled electron–photon wave function is constructed by binning random walkers at each time step into a set of equally sized histograms (i.e., shared by all timesteps) such that the creation or destruction of walkers does not affect the histogram binning. Normalization is enforced at the end of the simulation.

### 9.2 Auxiliary-Field QMC Method

We next consider the extension of auxiliary-field QMC (AFQMC) methods to electron-boson mixtures.^112,173^ Since the AFQMC formalism has been extensively applied in both quantum chemistry and condensed-matter physics, we do not detail its fundamentals here but instead refer interested readers to existing reviews. Our focus is on how AFQMC is adapted for electron-boson systems, highlighting the distinctions from conventional AFQMC for Fermions only. Two main strategies are commonly employed to project the coupled imaginary-time evolution operator. The first is a hybrid approach, combining AFQMC for electrons with DQMC for bosons in a first-quantized representation. The second approach treats both electrons and bosons via AFQMC in a purely second-quantized representation. In what follows, we outline these two methodologies and compare their respective advantages and limitations.

To perform QMC simulation of polaritons, we reorganize the one-body and two-body interactions in the so-called Monte Carlo (MC) format, i.e., rewriting the two-body interactions as the square of one-body operators so that Hubbard–Stratonovich transformations can be applied to transform the many-body interaction into the integral of one-body operators by introducing auxiliary fields.^174^ By using the modified Cholesky decomposition, the ERI can be rewritten as *Ṽ*_*pqrs*_ = ∑_γ_ *L*_*pq*,γ_^*e*^ *L*_*rs*,γ_^*e*,*^. Hence, the coulomb interaction can be rewritten as

$$\frac{1}{2}\sum_{\mathrm{pqrs}}^{M}{Ṽ}_{\mathrm{pqrs}}{ĉ}_{p}^{†}{ĉ}_{q}^{†}{ĉ}_{r}{ĉ}_{s}=-\frac{1}{2}\sum_{\mathrm{pq}}^{M}\sum_{r}L_{\mathrm{pr},\gamma }^{e}L_{\mathrm{qr},\gamma }^{e,*}{ĉ}_{p}^{†}{ĉ}_{q}+\frac{1}{2}\sum_{\gamma }\left(\sum_{\mathrm{pq}}L_{\mathrm{pq},\gamma }^{e}{ĉ}_{p}^{†}{ĉ}_{q}\right)\left(\sum_{\mathrm{rs}}L_{\mathrm{rs},\gamma }^{e,*}{ĉ}_{r}^{†}{ĉ}_{s}\right)$$

The first term on the right-hand side of above equation can be absorbed into the conventional kinetic operator. Consequently, the effective MC Hamiltonian for the polariton problem is

79 $${Ĥ}_{\mathrm{MC}}=\mathrm{T̂}+\frac{1}{2}\sum_{\gamma }{\mathrm{L̂}}_{\gamma }^{e,2}+\sum_{\alpha }\left[\left(\lambda_{\alpha }\cdot \mathrm{D̂}\right)\omega_{\alpha }{\mathrm{Q̂}}_{\alpha }+\frac{1}{2}(\lambda_{\alpha }\cdot \mathrm{D̂}{)}^{2}\right]+{Ĥ}_{\mathrm{ph}}$$

where the effective kinetic operator is *T̂* = ∑_*pq*_ *T*_*pq*_ *ĉ*_*p*_^†^ *ĉ*_*q*_ and $T_{\mathrm{pq}}=h_{\mathrm{pq}}-\frac{1}{2}\sum_{\lambda k}L_{\gamma ,\mathrm{ik}}^{*}L_{\gamma ,\mathrm{jk}}$ , and *L̂*_γ_ = *L*_*pq*,γ_^*e*^ *ĉ*_*p*_^†^ *ĉ*_*q*_. In addition, we can further decompose the bilinear term as

80 $$\sum_{\alpha }\left(\lambda_{\alpha }\cdot \mathrm{D̂}\right)\omega_{\alpha }{\mathrm{Q̂}}_{\alpha }=\frac{1}{4}\sum_{\alpha }\left[(\lambda_{\alpha }\cdot \mathrm{D̂}+\omega_{\alpha }{\mathrm{Q̂}}_{\alpha }{)}^{2}-(\lambda_{\alpha }\cdot \mathrm{D̂}-\omega_{\alpha }{\mathrm{Q̂}}_{\alpha }{)}^{2}\right]$$

The final MC format of the original PF Hamiltonian reads

81 $${Ĥ}_{\mathrm{MC}}=\mathrm{T̂}+\frac{1}{2}\sum_{\gamma }^{N_{\gamma }}{\mathrm{L̂}}_{\gamma }^{e,2}+\frac{1}{2}\sum_{\gamma^{\prime }}^{N_{\gamma \prime }}{\mathrm{L̃}}_{\gamma \prime }^{2}+{Ĥ}_{\mathrm{ph}}+C$$

where ${\mathrm{L̃}}_{\gamma \prime }\in \left\{\frac{\lambda_{\alpha }\cdot \mathrm{D̂}+\omega_{\alpha }{\mathrm{Q̂}}_{\alpha }}{\sqrt{2}},\frac{i\left(\lambda_{\alpha }\cdot \mathrm{D̂}-\omega_{\alpha }{\mathrm{Q̂}}_{\alpha }\right)}{\sqrt{2}}\right\}$ are the operators resulting from the decomposition of the bilinear coupling term.

#### 9.2.1 Trotterization and Hubbard–Stratonovich Transformation

To evaluate the imaginary time propagation, eq 68, in the CI space, we use Trotter decomposition to break the evolution operator into Trotter series,

82 $$e^{-\Delta \tau {Ĥ}_{\mathrm{MC}}}\approx e^{-\Delta \tau /2\mathrm{T̂}}e^{-\Delta \tau /2\Delta \tau {Ĥ}_{\mathrm{ph}}}e^{-\Delta \tau \sum_{\gamma }{\mathrm{L̂}}_{\gamma }^{2}/2}e^{-\Delta \tau \sum_{\gamma \prime }{\mathrm{L̃}}_{\gamma \prime }^{2}/2}e^{-\Delta \tau /2{Ĥ}_{\mathrm{ph}}}e^{-\Delta \tau /2\mathrm{T̂}}\times e^{-\Delta \tau C}+O\left(\Delta \tau^{3}\right)$$

Though the Trotterization introduces the Trotter error, high-order Trotter decomposition can be used to reduce the error. Besides, The Trotter error can be further reduced with an extrapolation procedure after separate calculations have been done with different values of Δ*τ*. The *H*_1_ part of the propagator is the exponential of a one-body operator. The *H*_2_ part is not. However, we can rewrite *e* ^–Δ*τH*_2_^ in the single-particle form via the Hubbard–Stratonovich (HS) transformation

83 $$e^{-\Delta \tau \sum_{\gamma }{\mathrm{L̂}}_{\gamma }^{2}/2}=\prod_{\gamma }\int dx_{\gamma }\frac{1}{\sqrt{2\pi }}e^{-x_{\gamma }^{2}/2}e^{x_{\gamma }\sqrt{-\Delta \tau }\mathrm{L̂}}$$

where {*x*_γ_} is the auxiliary field. The constant in front of *L̂* in the exponent on the right-hand side can be real or imaginary, depending on the sign of *L̂*. The key is that the quadratic form on the left is transformed into a high-dimensional integral over one-body propagators, parametrized by the auxiliary fields, which can be efficiently evaluated using Monte Carlo techniques.^175^ Regarding the bilinear term, the HS transformation of $e^{-\Delta \tau \sum_{\gamma \prime }{\mathrm{L̃}}_{\gamma \prime }^{2}/2}$ leads to the decoupled propagation of Fermionic and photonic components, as they commute. For example,

84 $$e^{x\lambda_{\alpha }\cdot \mathrm{D̂}+\omega_{\alpha }{\mathrm{Q̂}}_{\alpha }/\sqrt{2}}=e^{x\lambda_{\alpha }\cdot \mathrm{D̂}/\sqrt{2}}e^{x\omega_{\alpha }{\mathrm{Q̂}}_{\alpha }/\sqrt{2}}$$

Note that although we decouple the electronic and photonic degrees of freedom, they remain indirectly coupled via the shared auxiliary fields.^173^

#### 9.2.2 Mixed QMC Scheme for Polaritons

Within this scheme, the bosonic DOF is propagated via the DQMC scheme in the first quantization.^112^ Whereas, the boson-dressed electronic Hamiltonian is propagated within the AFQMC scheme. In particular, the bosonic WF is represented in the position state, which is the eigenstate of the bilinear coupling, making it straightforward to propagate the bilinear coupling term.

In Figure 6a and b, we showcase example calculations using determinant quantum Monte Carlo (DQMC) and auxiliary-field quantum Monte Carlo (AFQMC) methods for the H2 and hydrogen fluoride (HF) molecules, respectively. Both systems are sufficiently small to allow exact full configuration interaction (FCI) calculations, which serve as high-accuracy benchmarks for evaluating quantum Monte Carlo approaches under varying light–matter coupling strengths. We also compare the results with other quantum electrodynamical (QED) methods, including QED-Hartree–Fock (QED-HF) and QED-coupled cluster (QED-CC) approaches. In the case of the H2 molecule (Figure 6a), conventional CCSD yields exact results for this two-electron system. However, its QED extension (QED-CCSD-U22-S2), when truncated at a low photonic excitation level, introduces noticeable errors relative to the numerically exact DQMC results. This highlights the limitations of truncated QED-CC formulations in capturing strong light–matter correlations. Figure 6b presents results for the HF molecule, demonstrating the high accuracy of the QED-AFQMC method when compared to the exact QED-FCI (QED-FCI) reference. In contrast, the QED-CCSD-U22-S2 method yields qualitatively different energy behavior as a function of coupling strength, reflecting a failure to capture the correct topology of the energy landscape. This discrepancy stems from the simultaneous underestimation of both electron–electron and electron–photon correlations.^173^

**Figure 6:**
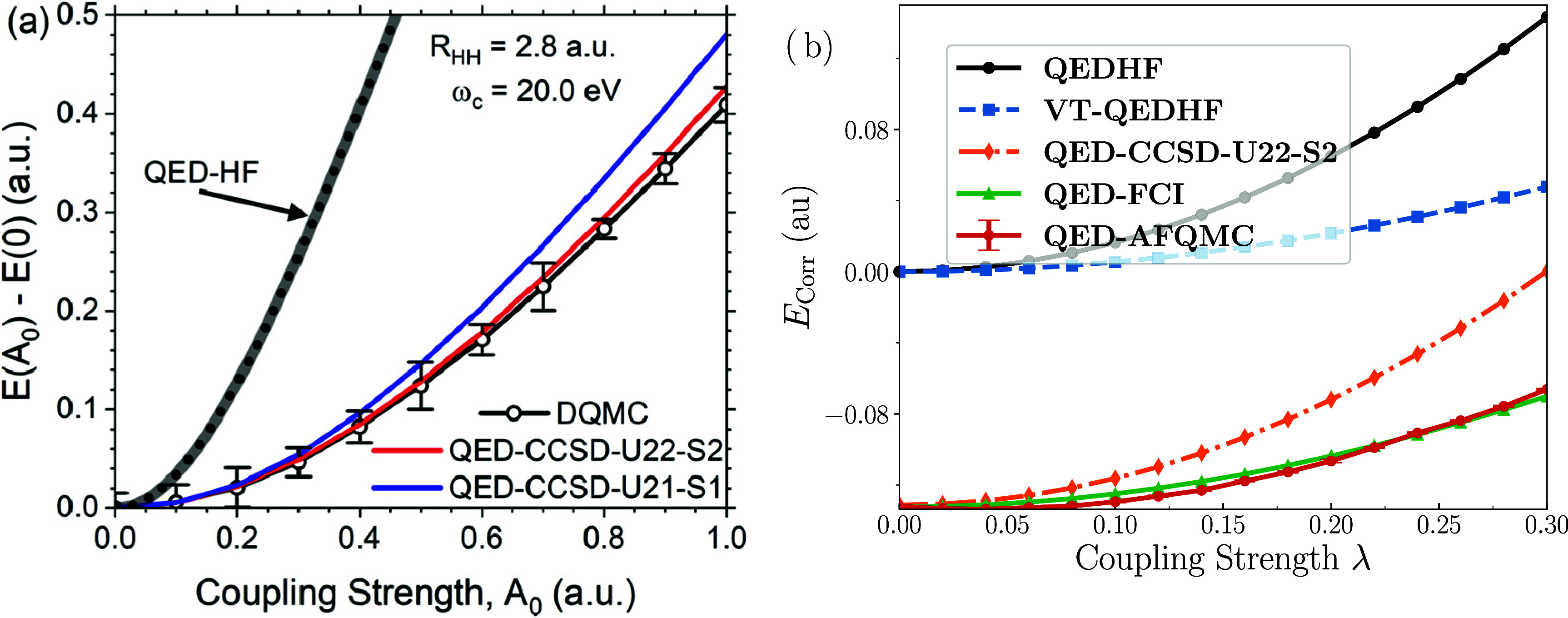
(a) The ground-state potential energy of the H2 system as a function of light–matter coupling strength. Though CCSD is exact for two-electron system, the QED-CCSD-U22-S2 underestimates the electron–photon correlations due to the truncation in low photonic excitations. (b) Correlation energies of the HF molecule as a function of electron–photon coupling strength λ computed from CCSD, FCI, and AFQMC. AFQMC exhibits excellent agreement with FCI across all λ, while CCSD systematically underestimates the correlation energy, particularly at stronger coupling strengths. Reproduced with permission from ref 64.

## 10 QED-DMRG

QED-CASCI is a powerful method for capturing strong correlation in cavity-coupled molecules. However, much like its conventional counterpart in quantum chemistry, it suffers from exponential scaling with the size of the active space, which typically limits practical applications to fewer than 20 strongly correlated orbitals. A natural route to overcome this limitation is to replace the FCI solver in the active space with a more scalable yet robust approximation that can still capture strong correlation. Two promising alternatives include methods based on two-electron reduced density matrices^176^ and those that use matrix product states (MPS) as the central ansatz. Here, we focus on the latter and describe the extension of DMRG to the Pauli–Fierz Hamiltonian for QED systems.^177–179^

The DMRG method is derived by reformulating the FCI wave function in the occupation number representation, where the full wave function is expressed as a rank-*k* tensor over *k* spin orbitals:

85 $$\left|\Psi_{\mathrm{FCI}}\right\rangle =\sum_{n_{1}\;\cdot \cdot \cdot n_{k}\;}c^{n_{1}n_{2}\cdot \cdot \cdot n_{k}}\left|n_{1}n_{2}\cdot \cdot \cdot n_{k}\right\rangle$$

Each occupation number *n*_*i*_ takes one of four values corresponding to the four possible occupations of a spatial orbital: empty, spin-up, spin-down, and doubly occupied.

In DMRG, this high-rank tensor is approximated by a product of lower-rank tensors in the MPS form:

86 $$\left|\Psi_{\mathrm{MPS}}\right\rangle =\sum_{n_{1}\cdot \cdot \cdot n_{k}}\sum_{i_{1}\cdot \cdot \cdot i_{k-1}}A{\left[1\right]}_{i_{1}}^{n_{1}}A{\left[2\right]}_{i_{1}i_{2}}^{n_{2}}\cdot \cdot \cdot A{\left[k\right]}_{i_{k-1}}^{n_{k}}\left|n_{1}\cdot \cdot \cdot n_{k}\right\rangle$$

The intermediate (virtual) indices *i*_*j*_ are contracted along the chain and truncated to a fixed maximum dimension. This truncation provides a controlled approximation to FCI that scales polynomially with system size for moderate entanglement.

The DMRG algorithm optimizes two neighboring tensors at a time in a sweeping procedure. At each step, the effective Hamiltonian for the selected sites is constructed by contracting the MPS tensors to the left and right to form renormalized block bases. These are then used to diagonalize the local Hamiltonian. After optimization, the basis is truncated by retaining the most significant eigenvectors of the reduced density matrix. Figure 7 illustrates this process in the standard and QED-extended cases.

**Figure 7:**
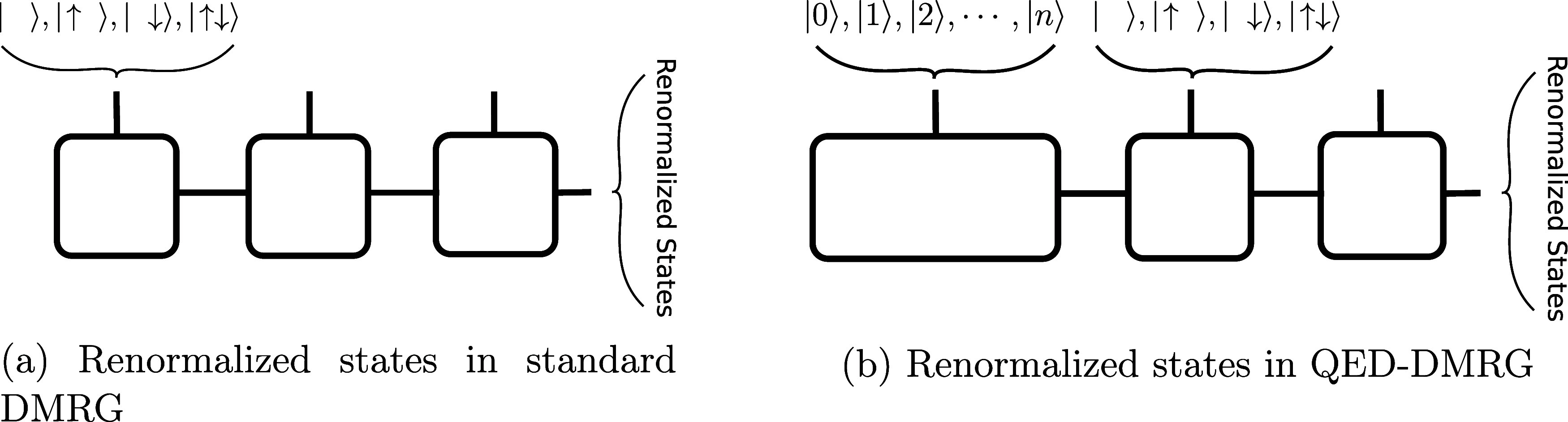
Renormalized states of the left block in DMRG obtained by partial contraction of the MPS chain, shown for both standard DMRG and QED-DMRG. Penrose tensor notation is used. These renormalized states form the basis in which the effective Hamiltonian is constructed.

The QED-DMRG extension was introduced by Matoušek and co-workers,^180^ who demonstrated its performance for oligoacene systems up to pentacene using an active space of 22 orbitals. This is far from the practical limit, and larger active spaces are expected as the implementation is further optimized. For reference, conventional DMRG applications in quantum chemistry have treated active spaces with over 76 orbitals,^181^ suggesting that polaritonic simulations of comparable scale are within reach.

The inclusion of the cavity mode into the DMRG framework is conceptually straightforward. A single MPS site is assigned to represent the photonic degree of freedom. Unlike electronic orbitals, this site has a larger local dimension, corresponding to the truncated photon Fock space {|0⟩, |1⟩,..., |*N*⟩}. The implementation requires additional bookkeeping to manage the bosonic ladder operators and their interactions with the Fermionic system, but otherwise integrates seamlessly into the DMRG sweep.

One significant advantage of QED-DMRG is its efficient handling of the photonic degree of freedom. The algorithm naturally optimizes out unnecessary components of the photon Hilbert space, and thus setting a high photon number cutoff does not incur a prohibitive cost—unlike in QED-CASCI, where the scaling is exponential.

Beyond computational efficiency, QED-DMRG provides access to powerful correlation diagnostics such as orbital mutual information. These measures, long employed within the DMRG community to optimize orbital ordering,^182^ also yield valuable insight into the entanglement structure of cavity-coupled molecules.^183^

An emerging frontier in QED chemistry is the study of heavy atoms and systems where relativistic effects become important. Cavity-induced spin-forbidden transitions, such as singlet–triplet couplings, may be significantly altered in heavy-element systems. DMRG has already been extended to relativistic Hamiltonians,^184,185^ and a natural next step would be to generalize these formulations to include photon coupling via the Pauli–Fierz Hamiltonian.

Vibronic effects are another key feature of polaritonic systems,^158^ and while in many cases these can be included using decoupled approaches based on potential energy surfaces,^74,186,187^ an exact treatment requires going beyond the Born–Oppenheimer approximation. DMRG methods based on the nuclear–electronic orbital (NEO) approach have been developed to treat electrons and nuclei on equal footing.^188,189^ Extending these NEO–DMRG formulations to incorporate photon modes is a promising future direction.

Despite its strengths, DMRG lacks dynamic correlation outside the active space. Several approaches address this limitation, including DMRG-PT2,^190^ pair-density functional theory (DMRG-PDFT),^191^ and adiabatic connection (AC)-based methods.^192^ Among these, AC is particularly attractive because it avoids the need for high-rank reduced density matrices (RDMs), which can otherwise scale poorly with active space size.^193^ Including dynamic correlation is essential for predictive accuracy, and the combination of QED-DMRG with AC is a compelling strategy to enhance the utility of this approach for quantitative polaritonic chemistry.^194^

## 11 Parametrized vs Self-Consistent QED Approaches

Approaches that bear conceptual similarity to QED-CI approaches are those which may be called projected or parametrized QED (pQED) approaches.^26,195–198^ As in QED-CI approaches, the fundamental idea is to express the coupled electronic-photonic states in a linear expansion of tensor products between electronic and photonic states. However, in contrast to the previously discussed self-consistent QED-CI approach in which the coupled states are directly solved for in a single step,^199^ the pQED approach follows a sequential two-step process. First, the matter degrees of freedom are computed using conventional electronic structure methods, and then the *ab initio* QED Hamiltonian is constructed in a linear expansion of basis states that are tensor products of electronic and photonic states.^26,198^

Both approaches are equivalent in the limit of complete orbital and many-electron basis, but outside this limit, the quadratic dipole self-energy term leads to a disparity, and the two approaches no longer agree.^197^ The projected dipole self-energy operator in pQED achieves exactness only in the complete orbital and many-electron basis limits, whereas scQED may be formulated using the exact form of the dipole self-energy operator in a specific orbital basis. To see this disparity, we consider a simple diatomic case where it is possible to approach both the complete orbital and many-electron basis simultaneously through both approaches, which is the HeH^+^ cation coupled to a cavity mode. This allows the use of scQED and pQED techniques to examine the behavior of the dipole self-energy on the ground state energy as we approach both the orbital and many-electron bases simultaneously. In this case, the scQED approach corresponds to QED-FCI­(10ph) in a given orbital basis (cc-pVXZ, with X = D, T, or Q), and the pQED approach utilizes adiabatic many-electron states arising from FCI in the same orbital basis. We denote the pQED results as pPF­(*N*_el_,10) since they correspond to the energies of the Pauli–Fierz Hamiltonian projected onto *N*_el_ many-electron states (from FCI) and 10 photonic Fock states. For each basis set, the absolute energy error of the ground state energy as computed by pPF­(*N*_el_,10) relative to QED-FCI-10, where *N*_el_ is the number of many-electron basis states used to parametrize the pPF Hamiltonian. It can be observed that the energy error decreases with increasing size of the orbital basis and with increasing *N*_el_ in a given orbital basis. In the comparable limit, the energy error of pPF­(*N*_el_,10)/cc-pVQZ converges to 0.1 microHartrees, whereas the energy error of pPF­(*N*_el_,10)/cc-pVDZ converges to ∼10 microHartrees when all FCI states are used to parametrize the Hamiltonian. In the limit that both the orbital and many-electron basis are complete, these results support the argument that the projected dipole self-energy approaches the exact dipole self-energy in the complete basis limit.^197^

In practical implementation, pQED and scQED diverge in their handling of the dipole self-energy. The treatment of the DSE term, particularly the quadratic electronic dipole term *d̂*_e_^2^, marks a crucial implementation difference between the pQED and scQED approaches. Although pQED involves specific state truncations, scQED maintains the exact form of electronic operators in its Hamiltonian formulation.^47^ So, the scQED Hamiltonian is written in terms of exact electronic operators. In first quantization, we can expand *d̂*_e_^2^ as

87 $${{\mathrm{d̂}}_{e}}^{2}=\sum_{i\neq j}d_{e}\left(i\right)d_{e}\left(j\right)+\sum_{i}{\left[d_{e}\left(i\right)\right]}^{2}$$

where *i* and *j* represent different electronic coordinates; From eq 85 the structure of the dipole self-energy operator shows the contribution from both a one-electron quadrupole-like term and a two-electron term. Manderna and co-workers recently showed that projection of the dipole self-energy operator in the pQED approach does not contain the one-electron quadrupole-like term.^197^ They also showed that this discrepancy only resolves under the simultaneous conditions that the projection of the dipole self-energy operator is performed onto the complete electronic Hilbert space, and that the operators are represented in a complete orbital basis. Outside of this limit, the pQED neglects this quadrupolar contribution to the dipole self-energy, leading to the energy errors shown in Figure 8. We note that these energy errors are quite small for the system under study, and it remains for future investigation to determine if the disparity between pQED and scQED approaches manifest in the predictive capabilities.

**Figure 8:**
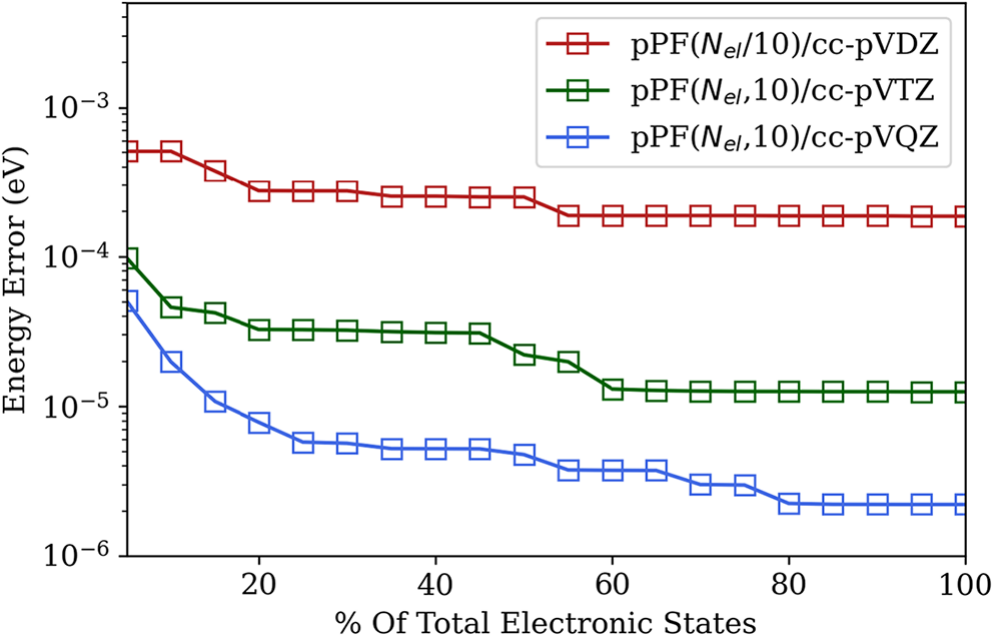
Absolute error of the pQED (*N*_el_,10) ground state energy relative to the scQED ground state energy computed within the cc-pVDZ, cc-pVTZ, and cc-pVQZ basis sets, where we plot this error as a function of the percentage of the FCI electronic states used in the parametrized method. The photon frequency in each case is tuned to the *S*_0_ → *S*_2_ transition, and λ_*z*_ is fixed at 0.02 atomic units. Reproduced with permission from ref 197.

## 12 QED Analytic Gradients and Hessians

Analytic nuclear gradients provide critical access to the geometry optimization and dynamical simulation of light–matter coupled systems at the *ab initio* level. Recent work has extended gradient theory to several many-body QED methods, enabling the evaluation of forces that explicitly account for the perturbed electron–photon interaction. Gradients have now been formulated and implemented for QED-DFT,^140^ QED-HF,^157,200^ and QED-CC,^157^ and have been used to probe cavity-induced structural changes in representative systems.

In the coherent-state formulation of QED-HF, the derivative of the total energy with respect to a nuclear or external perturbation χ takes the form

88 $$\frac{\partial E_{\mathrm{QED}-\mathrm{HF}}}{\partial \chi }=\frac{\partial E_{\mathrm{HF}}}{\partial \chi }-\frac{1}{2}\sum_{\mu \nu }q_{\mu \nu }^{\chi }\gamma_{\mu \nu }-\sum_{\mu \nu }\gamma_{\mu \nu }\sum_{\lambda \sigma }\gamma_{\lambda \sigma }d_{\mu \sigma }^{\chi }d_{\lambda \nu }$$

where $\frac{\partial E_{\mathrm{HF}}}{\partial \chi }$ is the conventional Hartree–Fock gradient, γ_*μν*_ is the one-particle density matrix, and the cavity-weighted derivative integrals are defined as

89 $$d_{\mu \nu }^{\chi }=\frac{\partial }{\partial \chi }\sum_{a\in \left\{x,y,z\right\}}\lambda_{a}\int \phi_{\mu }^{*}\left(-r_{a}\right)\phi_{\nu }d\tau$$

If the QED-DFT formulation employs a traditional exchange–correlation functional, the expression above remains valid, with the Hartree–Fock gradient replaced by the Kohn–Sham gradient. Otherwise, gradient contributions from the electron–photon exchange–correlation functional must also be included. Using this framework, Liebenthal and co-workers^140^ investigated orientation-dependent distortions in a model Diels–Alder reaction under strong coupling, and highlighted the need for caution when interpreting cavity-induced reactivity trends based solely on fixed geometries.

At the excited-state level, Shao and co-workers^57^ developed analytic gradients for QED-TDDFT. These expressions quantify how upper and lower polariton orbitals respond to nuclear or external perturbations, enabling the analysis of excited-state density redistribution in response to cavity effects. The gradients also track how photonic character transfers among nearby states, offering insight into state mixing in the strong-coupling regime.

Gradients for QED-CC were recently derived by Lexander and co-workers,^157^ who extended the coupled-cluster Lagrangian to include photonic amplitudes and their response. This generalization allows the QED-CC state to reach a variational stationary point with respect to the photon field. Their application to azobenzene revealed cavity-dependent variations in internal coordinates and highlighted how molecular structure may be altered by quantized light.

More recently, Barlini and co-workers^201^ derived analytic Hessians for QED-HF, extending the formalism to second-order response. This enabled the calculation of vibrational frequencies in cavity-coupled systems. Their results showed that vibrational modes previously inactive in the infrared spectrum could become optically active due to light–matter coupling, and that others shift in energy by tens of wavenumbers—underscoring the potential for cavity-modified vibronic structure.

## 13 Real-Time Methods

### 13.1 RT-QED-TDDFT

Malave and co-workers^202^ developed a real-space, real-time QED-TDDFT framework using a tensor product (TP) representation of electronic and photonic degrees of freedom. In this approach, the Kohn–Sham orbitals are discretized on a three-dimensional real-space grid, and the quantized photon field is expanded in a truncated Fock space basis. The total wave function is written as a hybrid real–Fock-space product:

90 $$\Psi \left(r_{1},...,r_{N};q_{\alpha },t\right)=\sum_{n_{\alpha }=0}^{N_{F}}\psi_{n_{\alpha }}\left(r_{1},...,r_{N},t\right)⊗\left|n_{\alpha }\right\rangle$$

where **r**_*i*_ are the coordinates of the *i*-th electron, *q*_α_ is the photonic displacement coordinate of mode α, and |*n*_α_⟩ denotes the Fock state with *n*_α_ photons in that mode. The truncation level *N*_*F*_ controls the maximum number of photon excitations retained in each mode.

Time propagation of the coupled electron–photon wave function is performed using a fourth-order Taylor expansion of the time-evolution operator:

91 $$\Psi \left(t+\Delta t\right)=\sum_{j=0}^{4}\frac{{\left(-i\Delta t\right)}^{j}}{j!}{Ĥ}^{j}\Psi \left(t\right)$$

where Δ*t* is the time step, *Ĥ* is the total system Hamiltonian, and *j* indexes the expansion order. This formulation, based on the Pauli–Fierz Hamiltonian in the length gauge and dipole approximation, captures both electronic dynamics and quantized light–matter interactions on equal footing.

The method grants access to a variety of quantum observables, including time-resolved photon number distributions, mode-resolved dipole moments, and cavity-induced Rabi splittings. Benchmark calculations on small molecules such as H_2_, LiH, and HF were shown to agree with QED-CC and stochastic variational methods. The authors further applied the framework to simulate dipole oscillations in Na_2_, orbital reshaping in benzene, and cavity-induced charge redistribution in donor–acceptor systems such as *p*-nitroaniline. In HF, they demonstrated cavity-modified high harmonic generation (HHG), where new spectral features emerged due to transitions mediated by quantized photon states. Although the computational scaling is exponential in the number of photon modes *N*_*p*_ and Fock truncation *N*_*F*_, i.e., $O\left(N_{F}^{N_{p}}\right)$ , the method provides a numerically exact and systematically improvable approach for simulating strongly coupled cavity–molecule dynamics in systems where a small number of photon modes dominate the interaction.

More recently, Bonafé and co-workers extended the Maxwell–Pauli–Kohn–Sham framework^203^ to treat beyond-dipole light–matter interactions using a fully minimal coupling Hamiltonian.^204^ Their approach couples the real-space Maxwell equations to the time-dependent Kohn–Sham equations via the full space- and time-dependent electromagnetic vector potential, providing a nonperturbative and gauge-consistent treatment of radiative feedback. By avoiding multipolar truncations and enabling spatially inhomogeneous fields, this framework captures phenomena inaccessible to dipole-limited approximations. Applications include renormalized Cherenkov radiation, magneto-optical activity in achiral systems, and field-induced frequency shifts in plasmonic nanostructures. This work represents a substantial advance toward general-purpose, real-space quantum electrodynamics simulations of light–matter dynamics in strongly inhomogeneous environments.

### 13.2 QED-TDCI

The QED-TDCI method simulates the time evolution of a mixed cavity-molecule wave function subject to a time-dependent external electric field (e.g., a laser pulse). The total Hamiltonian consists of a time-independent term *Ĥ*_cm_ describing the cavity-molecule interaction and a time-dependent electric field term *Ĥ*_las_

92 $$Ĥ\left(t\right)={Ĥ}_{\mathrm{cm}}+{Ĥ}_{\mathrm{las}}\left(t\right)$$

The laser field is treated semiclassically in the length gauge and long wavelength approximation as

93 $${Ĥ}_{\mathrm{las}}\left(t\right)=-{\mathrm{\mu ̂}}_{\mathrm{cm}}\cdot F\left(t\right)$$

The time evolution is performed in the basis of *Ĥ*_cm_ eigenstates, meaning that the matrix form of *Ĥ*_cm_ in eq 92 is a diagonal matrix containing the polaritonic state energies. The *Ĥ*_las_ term is in general not diagonal, but contains off-diagonal terms describing the coupling between states arising from the dipole operator. Performing the time propagation in this way means that the time-dependent part is relatively inexpensive, and the bottleneck is generally the obtaining of the cavity-molecule eigenstates and transition dipole matrix elements.

The cavity-molecule eigenstates for QED-TDCI can, in principle, be obtained with any QED-CI excited state method. Here, we discuss QED-TDCI as originally formulated in refs,^205,206^ wherein the pQED-CI method (Section 11) is used to solve the PF Hamiltonian of eq 5. The pQED-CI method was chosen for use with QED-TDCI for three reasons. First, it allows for a simple interpretation of the polaritonic eigenstates in terms of the uncoupled light and matter states. Second, it enables rapid prototyping across different regimes of cavity parameters. Because the electronic structure portion remains constant in pQED, recomputing *Ĥ*_cm_ with different cavity parameters (coupling strength, frequency, polarization direction) requires modifying only the photonic terms in *Ĥ*_cm_, and not recomputing the earlier electronic structure steps. Lastly, the TDCI scheme as presented requires many excited states in the time-independent basis, a requirement naturally met by the pQED-CI framework. The alternative scQED approach, as mentioned in Section 11, would instead be well-suited to the alternative time-dependent CI methodology proposed by Levine and co-workers,^207^ which propagates the wave function directly in the orbital basis rather than propagating the coefficients of an expansion of time-independent states. The limitations of truncation in the pQED-CI approach, particularly in the disparity between the projected and exact dipole self-energy operator, for dynamic properties is the topic of a future study.

The procedure to perform a QED-TDCI computation based on pQED-CI is as follows. First, a set of electronic eigenstates and corresponding transition dipole matrix elements are solved using traditional CI electronic structure methods. We note that the pQED-CI method is agnostic to the manner of CI used, so methods such as CIS, CASCI, or even full CI can be substituted depending on the desired level of correlation to include for the molecular system of interest. This feature has the convenient advantage of being able to use existing electronic structure packages to perform the electronic portion of the computation.

Next, the PF Hamiltonian (eq 5) is constructed in the cavity-molecule direct product basis, forming a block-tridiagonal matrix.^205^ This matrix is diagonalized to obtain the polaritonic eigenstates. The dipole moment operator in the cavity-molecule eigenstate basis, μ̂_cm_, must be constructed for both the laser field in eq 91 and the calculation of the time-dependent dipole moment and related properties. The total dipole moment operator of the polaritonic system is a product between the electronic and photonic dipole operators, μ̂^*tot*^ = μ̂_*e*_ ⊗ μ̂_*p*_ (suppressing vector and coordinate notation for brevity). For polaritonic states *p*,*q*,..., electronic states *a*,*b*,..., and photonic states α,β,..., the transition dipole matrix elements in the electron–photon direct product basis μ̃ are computed as

94 $$\mathrm{\mu ̃}=\left\langle p\right|{\mathrm{\mu ̂}}^{\mathrm{tot}}\left|q\right\rangle =\left\langle \alpha a\right|\left[{\mathrm{\mu ̂}}_{e}⊗{\mathrm{\mu ̂}}_{p}\right]\left|b\beta \right\rangle$$

The photonic contribution, μ̂_*p*_, is related to the photonic displacement coordinate.^71^ However, due to its dependence on experimental parameters and a focus on the cavity influence on the *molecular* properties, this term is generally ignored and can replaced by the identity matrix (see ref 196 and references therein for a more detailed discussion). The electronic transition dipole matrix in the electron–photon basis then becomes (for two photonic basis states)^205^

95 $$\mathrm{\mu ̃}=\left(\begin{array}{ll} D_{\mathrm{CI}} & 0\\ 0 & D_{\mathrm{CI}} \end{array}\right)$$

where **D**_CI_ is the electronic transition dipole matrix in the CI eigenstate basis. Finally, μ̃ is rotated into the cavity-molecule basis, using the eigenvectors of *Ĥ*_cm_.^205^

The time propagation in QED-TDCI is performed analogously to the standard electron-only TDCI approach, except that the basis of the propagation is pQED-CI eigenstates. The form of the laser field is typically taken to be a cosine squared envelope with a cosine carrier frequency,

96 $$F\left(t\right)=\epsilon_{l}A\cos^{2}\left(\frac{\pi }{2\sigma }\left(t-t_{0}\right)\right)\cos\left(\omega_{l}\left(t-t_{0}\right)\right)$$

where ϵ_*l*_ is a unit vector describing the polarization direction, *A* is the maximum field strength, σ is the full-width at half-maximum, ω_*l*_ is the frequency, and *t*_0_ is the time for the center of the pulse. Other pulse forms, such as Gaussians or chirped pulses, may also be utilized to promote targeted excitations.^208^

The time-dependent wave function is expanded in the basis of time-independent pQED-CI eigenstates,

97 $$\left|\Psi \left(t\right)\right\rangle =\sum_{n}^{N}C_{n}\left(t\right)\left|\Psi_{n}\right\rangle$$

with the time-dependence carried by the coefficients *C*_*n*_, and the sum is over all polaritonic states included in the simulation. A time-evolution operator, *Û*(*t*_0_, *t*), maps |Ψ­(*t*_0_)⟩ into |Ψ­(*t*)⟩, viz.

98 $$\left|\Psi \left(t\right)\right\rangle =Û\left(t_{0},t\right)\left|\Psi \left(t_{0}\right)\right\rangle$$

Propagation proceeds according to the electronic TDCI procedure, *i.e., Û*(*t*, *t* + Δ*t*) = *e* ^–*iĤ*(*t*)Δ*t*^. Substitution of eq 95 into the time-dependent Schrödinger equation provides the time-dependence of the coefficients

99 $$C_{n}\left(t+\Delta t\right)=e^{-iĤ\left(t\right)\Delta t}C_{n}\left(t\right)$$

The QED-TDCI method has been used to investigate the dynamics of state transitions in the strong coupling regime under the effects of shaped pulse sequences.^205,206^ These pulse shapes are determined using the π-pulse condition,^209^ which produces the optimal pulse to produce a state transition for a two-state system in the rotating wave approximation. For a small test system, the LiCN molecule, paths for driving state populations from the ground state into the LP and UP were described.^205^ Specifically, the strong electric dipole coupling between the LP and UP can be utilized to rapidly drive population to the UP following excitation into the LP from the ground state. Figure 9a–c shows the state populations, electric field pulses, and resulting time-dependent dipole moment for a two-pulse sequence, showcasing controlled dipole-switching dynamics at the femtosecond time scale.

**Figure 9:**
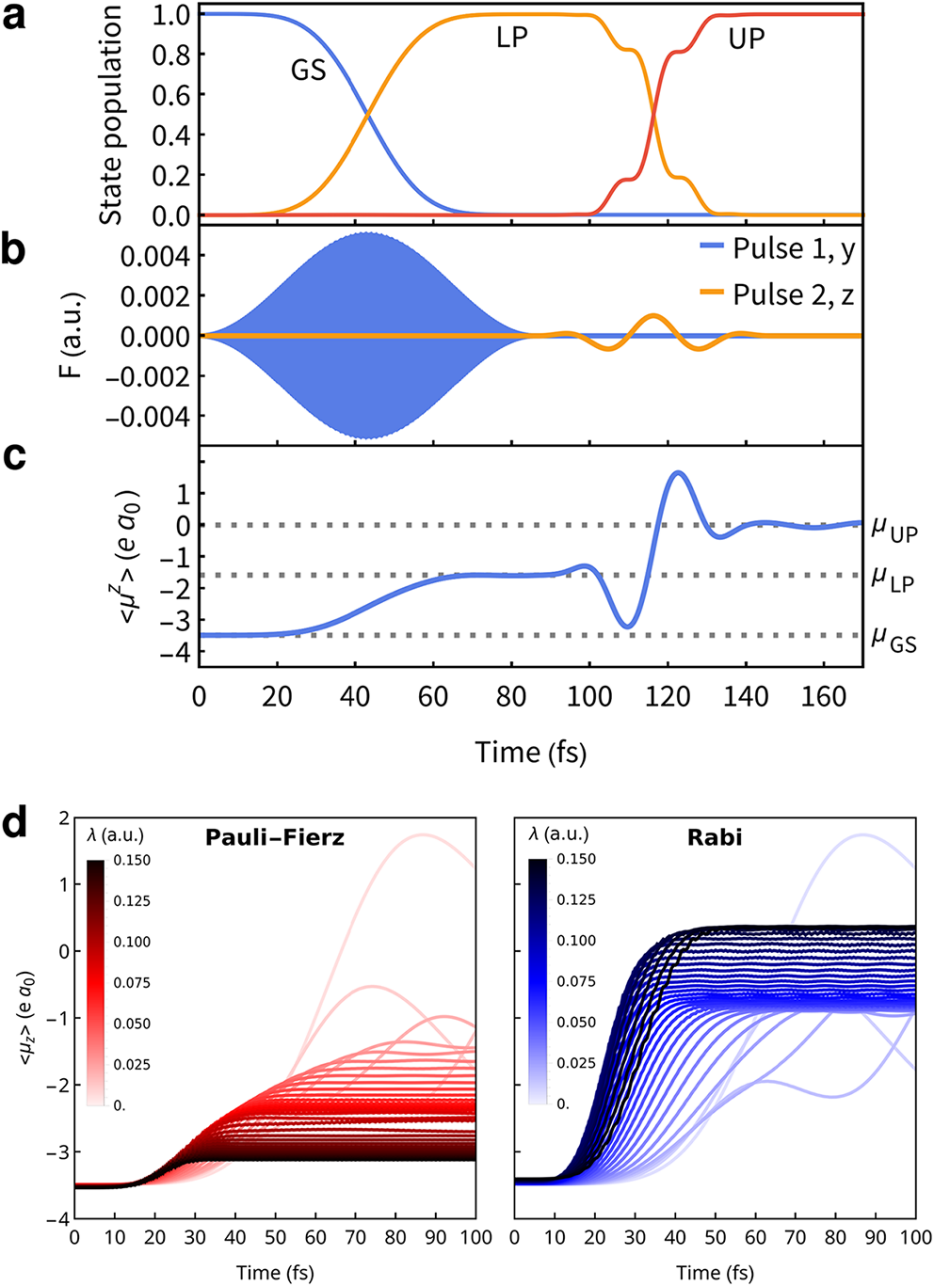
(a–c) Reprinted from ref 205, with the permission of AIP Publishing. (d) Adapted with permission from ref 206 Optica Publishing Group. Dynamics of LiCN molecule in a cavity using QED-TDCI. (a–c) Demonstration of polaritonic multistate hopping between the ground state (GS) lower polariton (LP) and upper polariton (UP) using a sequence of two laser pulses. (a) State populations (b) Electric field strength for the two pulses (c) Expectation value of the electronic dipole moment along the *z* axis. The permanent *z* dipole moments of the states are indicated by dotted lines. (d) Time-dependent expectation value of the electronic dipole moment as a function of cavity-molecule coupling strength (λ) for the (left) Pauli–Fierz and (right) Rabi model Hamiltonians. For each value of λ, a QED-TDCI simulation was performed with a π-pulse targeting the GS-LP transition.

Additionally, thanks to the flexible nature of the pQED scheme adopted, determining contributions from the individual coupling terms in the PF Hamiltonian in eq 5 is possible without repeated calculation of the underlying electronic states.^206^ The Rabi Hamiltonian, for example, may be produced by simply neglecting the dipole self-energy term. To assess the importance of this term for dynamic properties, Figure 9d shows the difference between the time-dependent dipole moments for the LiCN molecule under a π-pulse using the Pauli–Fierz (top) and Rabi (bottom) model Hamiltonians. These data show that for even modest coupling strengths, the dipole self-energy term is critical for computing accurate dynamics. At very strong coupling, the sign of the dipole moment following the electric field pulse is incorrect using the Rabi Hamiltonian. This suggests that the Rabi Hamiltonian is insufficient for even qualitative work in the strong coupling regime.

### 13.3 RT-QED-CC

The QED-CC method described in Section 8 has also been extended to the time-domain by Koch and co-workers,^210^ implemented in a development version of the $e^{T}$ program package.^211^ The time-dependent wave function is parametrized using time-dependent cluster operators *T̂*(*t*) acting on a QED-HF reference in the coherent-state representation, |HF,0⟩, with a global phase α­(*t*),

100 $$\left|\Psi_{\mathrm{RT}‐\mathrm{QED}‐\mathrm{CC}}\left(t\right)\right\rangle =e^{\mathrm{T̂}\left(t\right)}\left|\mathrm{HF},0\right\rangle e^{i\alpha \left(t\right)}$$

Like TDCI, this is generally done in the presence of a time-dependent, classical electric field shaped by an envelope function; however, the method was also validated via imaginary time propagation. As in electronic real-time coupled cluster methods,^212–219^ differential equations for the time-dependent amplitudes in eq 100 are obtained via projection of the time-dependent Schrödinger equation, e.g., for the RT-QED-CCSD-1 model:

101a $$\frac{dt_{\mu }}{dt}=-i\left\langle \mu ,0\right|e^{-\mathrm{T̂}\left(t\right)}\left(\mathrm{H̃}+Ṽ\left(t\right)\right)e^{\mathrm{T̂}\left(t\right)}\left|\mathrm{HF},0\right\rangle$$

101b $$\frac{ds_{\mu }}{dt}=-i\left\langle \mu ,1\right|e^{-\mathrm{T̂}\left(t\right)}\left(\mathrm{H̃}+Ṽ\left(t\right)\right)e^{\mathrm{T̂}\left(t\right)}\left|\mathrm{HF},0\right\rangle$$

101c $$\frac{d\gamma }{dt}=-i\left\langle \mathrm{HF},1\right|e^{-\mathrm{T̂}\left(t\right)}\left(\mathrm{H̃}+Ṽ\left(t\right)\right)e^{\mathrm{T̂}\left(t\right)}\left|\mathrm{HF},0\right\rangle$$

101d $$\frac{d\alpha }{dt}=-\left\langle \mathrm{HF},0\right|\left(\mathrm{H̃}+Ṽ\left(t\right)\right)e^{\mathrm{T̂}\left(t\right)}\left|\mathrm{HF},0\right\rangle$$

The resulting amplitude expressions are then numerically propagated using e.g., the Runge–Kutta class of numerical integrators.^220^ As in TDCI, the time-dependence of observables (such as the electric dipole moment) may be evaluated by computing their expectation values at each time step. Since the QED-CC Hamiltonian is non-Hermitian, this requires propagation of distinct left-hand wave function amplitudes, which are derived and propagated in an analogous fashion to eqs 101a–101d.

The RT-QED-CCSD method was used to model charge migration dynamics in a perpendicular H_2_ dimer and the succinic semialdehyde molecule. By separating two perpendicular H_2_ monomers in space (up to 50 Å), couplings via the electronic and photonic degrees of freedom were effectively isolated. After the application of a short electric field pulse, energy transfer between the two hydrogen molecules was demonstrated, which was not present outside the cavity. An analysis of the time-dependent induced dipole moment and photon coordinate revealed that excitations from the first hydrogen molecule into the photon field, which then produced excitations in the second hydrogen molecule, were responsible for this energy transfer. Periodicity in the oscillations of both the dipole moment and photon coordinate can be traced back to the energetics of the polaritonic states, resulting in quantum beats. These beats can be controlled by tuning the bond length of a single H_2_, introducing asymmetry and therefore additional unique time scales.

Intramolecular charge migration in succinic semialdehyde revealed complex mediation of the excited state dynamics through cavity coupling. Electronic states which were shown to be relatively unimportant outside the cavity contributed to significant modifications of the time-dependent electric dipole moment inside the cavity. Coupling to the cavity mode promoted energy redistribution from the acidic group on one end of the molecule to the aldehyde moiety on the opposite side. Depending on the electric transition dipole moment, the photon field may prioritize one direction or another, which is evidenced by inspecting the density displacement and induced electric dipole moment across the molecule. Together, these results showcase the potential for RT-QED-CC to accurately describe real-time polariton dynamics in chemical systems, particularly energy and charge migration phenomena, as well as the complex interplay between electronic and photonic degrees of freedom inside a cavity.

## 14 Coupled Electrons, Photons, and Nuclei

While the previous sections have focused on describing the strongly coupled electronic and photonic degrees of freedom in an exciton-polariton, the impact on nuclear degrees of freedom is often central to the phenomena observed. The experiments that rekindled the resurgence in cavity-QED highlighted suppression in chemical reaction rates when molecules were put in resonant cavities.^16^ Since electronic frequencies vary with nuclear configuration, their closeness to the cavity mode frequency varies, and consequently, the effective strength of the electron–photon coupling, so the potential energy surfaces that determine the motion of the nuclei are distorted away from their uncoupled Born–Oppenheimer shapes, thus altering reaction rates. On the other hand, in vibrational strong-coupling, the nuclei take over from the electrons in playing the fundamental role in their coupling to the photon modes.

Thus, a full understanding of polaritonic phenomena requires a description of coupled electrons, photons, and nuclei. Clearly, a direct application of the time-dependent Schrödinger equation for the propagation of the combined photonic-electronic-nuclear degrees of freedom is computationally intractable except for the smallest systems.^221–227^ While these studies have been instructive to highlight different novel features of electron–photon-nuclear correlation, it is imperative to model more realistic systems to be able to make definitive interpretations about the physics observed in the experiments.

To this end, mixed quantum-classical methods developed in the chemical dynamics community for coupled electron–nuclear dynamics have been extended to the polaritonics case. Typically, the nuclei are treated via classical trajectories, and the Born–Oppenheimer potential energy surfaces that form the landscape for electron–nuclear dynamics are replaced by polaritonic surfaces.^228,229^ These are defined by eigenstates of *Ĥ*_pol_ which denotes the full Pauli–Fierz Hamiltonian with the nuclear kinetic energy subtracted. It is useful at this point to give the Pauli–Fierz Hamiltonian, in the long-wavelength approximation in the length gauge, including the nuclear degrees of freedom, in first-quantization:

102 $${Ĥ}^{\mathrm{PF}}={\mathrm{T̂}}_{n}+{Ĥ}_{\mathrm{pol}},{Ĥ}_{\mathrm{pol}}={Ĥ}_{BO}+{Ĥ}_{p}+{\mathrm{V̂}}_{\mathrm{pm}}+{\mathrm{V̂}}_{SP}$$

where *T̂*_*n*_ = −∑_*I*_^*N*_*n*_^∇_*I*_^2^/(2*M*_*I*_) is the nuclear kinetic energy, *Ĥ*_BO_ is the Born–Oppenheimer Hamiltonian of the molecule, ${Ĥ}_{p}=\frac{1}{2}\sum_{\alpha }\left({\mathrm{p̂}}_{\alpha }^{2}+\omega_{\alpha }^{2}{\mathrm{q̂}}_{\alpha }^{2}\right)$ is the free photon field, *V̂*_*pm*_ = ∑_α_ω_α_ *q̂*_α_λ_α_·(∑_*I*_^*N*_*n*_^ *Z*_*I*_ **R̂**_*I*_ – ∑_*i*_^*N*_*e*_^ **r̂**_*i*_) is the bilinear photon-matter coupling, and ${\mathrm{V̂}}_{\mathrm{SP}}=\frac{1}{2}\sum_{\alpha }{\left(\lambda_{\alpha }\cdot \left(\sum_{I}^{N_{n}}Z_{I}{\mathrm{R̂}}_{I}-\sum_{i}^{N_{e}}{\mathrm{r̂}}_{i}\right)\right)}^{2}$ the self-polarization term. In the mixed quantum-classical methods, the nuclear subsystem is treated classically, with each classical nuclear trajectory associated with a time-dependent electron–photon wave function, often expanded in terms of polaritonic eigenstates.

Finding polaritonic surfaces via diagonalization in the combined electron–photon Hilbert space becomes rapidly inefficient when many photon modes need to be included in the simulation. QEDFT,^53,70,71^ cumulant expansions,^230^ tensor networks,^231^ and quasi-normal modes,^232^ accounting also for dissipation, have been developed to treat this situation. Inspired by the classical treatment of nuclear degrees of freedom, the cavity multitrajectory Ehrenfest method has been applied to treat hundreds of photon modes, as is further discussed in Section 14.3.

In methods that treat either the nuclei and/or the photons via classical trajectories, a natural question that arises is how well does the underlying potential for the classical entity capture the correlation with the quantum system, and how well does the classical description capture coupling terms that influence the quantum subsystem. A precise answer to this question is given by the exact factorization approach in which the exact wave function for the fully quantum electron–photon-nuclear system is factorized into a marginal part depending on some of the degrees of freedom and a conditional part.^233,234^ The equation that the marginal part satisfies has a Schrödinger form, with a time-dependent vector potential and scalar potential that depend on the conditional part, and that incorporates completely and exactly the coupling to the other degrees of freedom. The exact factorization approach has been extended to include photons,^235,236^ and this is further discussed in Section 14.4.

### 14.1 Non-Adiabatic Dynamics in the Polaritonic Landscape

Recent progress in molecular cavity quantum electrodynamics has enabled increasingly realistic simulations of how strong light–matter coupling modifies chemical reactivity. Notably, Hu and Huo have developed an *ab initio* trajectory surface hopping (TSH) framework that combines the Pauli–Fierz Hamiltonian with on-the-fly CASSCF electronic structure calculations, allowing for direct simulation of nonadiabatic polariton dynamics in molecules like azomethane.^195^ Nuclear forces are rigorously derived using polariton-state gradients, and cavity loss is treated through Lindblad dynamics, enabling a realistic description of dissipative hybrid light–matter dynamics.

These advances build upon foundational efforts by Groenhof and collaborators, who introduced a multiscale QM/MM molecular dynamics framework to simulate photoactive molecules strongly coupled to cavity modes.^237^ Their work enabled simulations of polaritonic effects in large molecular ensembles and provided a bridge between microscopic chemical dynamics and macroscopic cavity field behavior. This framework has since been extended to study surface hopping under strong coupling using semiempirical electronic structure methods such as AM1/FOMO-CI,^238^ and to explore the impact of cavity losses through non-Hermitian formulations.^239^

Earlier studies also employed Ehrenfest dynamics to describe coupled electronic–nuclear–photonic motion due to its simplicity and mean-field formulation.^54^ However, Ehrenfest methods inherently average over adiabatic surfaces and fail to capture stochastic transitions and decoherence, which are essential for accurate simulation of processes such as isomerization or conical intersection branching. These limitations are now well recognized, with more accurate trajectory-based methods like TSH being preferred for polaritonic nonadiabatic dynamics.^240^

A key remaining bottleneck is the lack of dipole derivatives, especially transition dipoles, in correlated wave function methods such as CASSCF or MRCI. These are essential for evaluating nuclear gradients of polariton surfaces and light–matter couplings. As a future direction, machine learning (ML) offers a practical solution. Hu and Huo have shown that kernel ridge regression models trained on *ab initio* dipole data can predict both dipoles and their derivatives with high fidelity,^195^ enabling fully energy-conserving dynamics while avoiding the need for analytic dipole gradients. These ML-enhanced simulations provide a promising path toward scalable, chemically accurate polaritonic dynamics in complex systems.

### 14.2 Collective Strong-Coupling: Many Molecules

Changes to physicochemical properties of materials have so far only been observed when very many material excitations are collectively coupled to the confined light modes of an optical resonator. Although recent theoretical work of Ruggenthaler, Rubio and co-workers suggest that collective strong coupling can locally perturb the polarizability of a single molecule,^34,78,241^ which in principle could be captured with the single-molecule approaches outlined so far in this perspective, it remains unknown if, or how, such polarizability changes affect the molecular reactivity in electronic ground or excited states, or enhance the transport of excitons and charges between molecules.^6,8,9,242^ Therefore, in addition to single-molecule approaches for describing chemistry in the strong coupling regime, also methods that account for the collective coupling of a large number of molecules, are needed to advance our understanding of how strong coupling affects the dynamics in excited electronic states. To keep simulations computationally tractable, additional approximations are, however, necessary. Because the enhancement of the vacuum field strengths is typically rather small in Fabry-Pérot cavity or plasmonic surfaces and lattices, describing a self-consistent approach to light–matter coupling can be avoided in favor of a divide-and-conquer scheme, in which the matrix elements of the light–matter Hamiltonian are computed in a basis of product states between uncoupled molecular states obtained from a ordinary quantum chemistry calculation, and Fock states of cavity modes.^237,243^ If direct coupling between the molecules are also neglected, the quantum chemistry calculations are independent, and this scheme becomes trivially parallel. Indeed, systems with thousands of molecules have been simulated to unravel how collective strong coupling affects the transfer of excitation energy between molecules.^6,244–247^ The results of such simulations emphasize the critical role of molecular vibrations in polariton-enhanced exciton transport. By driving nonadiabatic transitions between the delocalized and propagating bright polaritonic states on the one hand, and the localized and stationary dark states on the other hand,^248,249^ these vibrations renormalize the polaritonic group velocities for in-plane propagation, in line with experimental observations.^3,8,242^ Recently, a framework for simulating an arbitrarily large ensemble of molecules coupled to realistic cavity fields was proposed, that course-grains subsets of molecules into one or more supermolecule, each effectively representing the dynamics of many identical molecules,^250^ at the expense of reduced disorder; intramolecular proton transfer, polariton relaxation, and exciton transport were studied.

### 14.3 Treating Many Photon Modes

A numerically exact solution of the TDSE can be achieved by direct diagonalization of the full Hamiltonian. Already, the diagonalization of the electronic Hamiltonian is feasible for only a small number of degrees of freedom, and including also the Fock states of the photonic part, means that the coupled problem is infeasible to diagonalize beyond more than a few photon modes.

Fortunately, a quasi-classical description of quantum dynamics allows for scalable methods that incorporate multiple photonic degrees of freedom in a semiclassical manner. One such scheme is the cavity Multi-Trajectory Ehrenfest (cMTE) method.^251,252^

When investigating polaritonic phenomena typically “dark” cavities are investigated where the photon field is initially in its ground state. The corresponding ground-state wave function Ψ_0_(*q*) is positive-definite, and thus the probability distribution of the photon field at temperature *T* = 0 can be obtained without approximation through a Wigner transformation given by

103 $$W\left(q,p\right)=\frac{1}{2\pi \hbar }\int dy\exp\left(-\frac{\mathrm{ipy}}{\hbar }\right)\Psi_{0}\left(q+\frac{y}{2}\right)\Psi_{0}^{*}\left(q-\frac{y}{2}\right)$$

Under the assumption that the photon (P) and matter (M) subsystems are initially factorizable, the total density matrix ρ­(*X*, *t*) at time *t* = 0 is given by

104 $$\mathrm{\rho ̂}\left(X,t=0\right)={\mathrm{\rho ̂}}_{M}\left(t=0\right)\rho_{P,W}\left(X,t=0\right)$$

The respective subsystems can be found by tracing out the other degrees of freedom, i.e.,

105a $${\mathrm{\rho ̂}}_{M}\left(t\right)={\mathrm{Tr}}_{P}\left(\mathrm{\rho ̂}\left(X,t\right)\right)$$

105b $$\rho_{P,W}\left(X,t\right)={\mathrm{Tr}}_{M}\left(\mathrm{\rho ̂}\left(X,t\right)\right)$$

The Wigner distribution of the photon modes at *t* = 0 reads

106 $$\rho_{P,W}\left(X,0\right)=\prod_{\alpha }\frac{1}{\pi }\exp\left[-\frac{P_{\alpha }^{2}}{\omega_{\alpha }}-\omega_{\alpha }Q_{\alpha }^{2}\right]$$

The initial coordinates and momenta of the photon modes are thus sampled from this probability distribution ρ_*P,W*_(*X*, 0).^253,254^

Each initial point {*Q*_α_, *P*_α_} can then be evolved independently according to classical equations of motion governed by the Ehrenfest Hamiltonian *H*_*P*,*W*_

107 $$\frac{dQ_{\alpha }}{dt}=\frac{\partial H_{P,W}}{\partial P_{\alpha }},,\frac{dP_{\alpha }}{dt}=-\frac{\partial H_{P,W}}{\partial Q_{\alpha }}$$

The evolution of the probability distribution of the photon field ρ̂_*P*,*W*_(*X*, *t*) is therefore represented as a statistical ensemble of the evolution of these independent trajectories. The Ehrenfest equations of motion for the matter system are

108 $$\partial_{t}{\mathrm{\rho ̂}}_{M}\left(t\right)=-i\left[{Ĥ}_{M}+{Ĥ}_{\mathrm{MP},W}\left(X\left(t\right)\right),{\mathrm{\rho ̂}}_{M}\left(t\right)\right]$$

where *Ĥ*_*MP*,*W*_ denotes the Wigner-transformed coupled Hamiltonian, and *Ĥ*_*M*_ is the matter Hamiltonian. Observables are computed by averaging over the ensemble of trajectories and normalizing by the total number of trajectories.

The cMTE method offers several interesting advantages for modeling light–matter interactions. Unlike methods relying on a Fock-state basis, the cMTE method is not restricted to specific photon number subspaces, enabling a scalable semiclassical description of multiple photon modes as well as the inclusion of multiple photons. Prior studies include up to 400 photon modes coupled to different atomic systems.^251,252^ In addition to its ability to treat a large number of photon modes, the cMTE method provides a computationally efficient and accurate description of key quantum optics phenomena, such as spontaneous emission, single- and two-photon emission, bound photon states, and *g* ^2^ autocorrelation functions.^251^

While the cMTE method presented here treats electronic and photonic degrees of freedom, the cMTE method can be trivially extended to nuclear degrees of freedom. Ref 255 employed such an extension to study 70 photon modes coupled with explicit nuclear degrees of freedom. This enabled the capturing of cavity-induced suppression of proton-coupled electron transfers. It reveals the impact of multimode photon cavities and self-polarization on photochemical suppression.^255^

Ehrenfest dynamics, while rigorously derived from the quantum-classical Liouville equation, still only captures semiclassical effects. As a result, “zero-point energy leakage” which describes the unphysical energy leakage is a known problem of Ehrenfest dynamics. Ref 256 addresses this by introducing an ad-hoc correction through the addition of classical coordinates which explicitly represent vacuum fluctuations. The interaction of the light with these auxiliary coordinates is scaled according to the instantaneous ground-state population.

A formal justification of Ehrenfest dynamics is only valid for a harmonic photonic potential with a bilinear coupling term. Therefore, another challenge arises from the feedback from the matter to the light subsystem, which introduces anharmonicities in the photonic potentials.^236^ A detailed analysis using the exact factorization framework is provided in ref 257.

The cMTE method is part of a wider effort to study light–matter interactions (semi)­classically. There are a number of variations to the cMTE method. First, while the scalability with respect to the number of photon modes is a key advantage of the cMTE method, similar approaches have been explored in single-mode systems.^258^

Second, the cMTE method adopts a quantum-mechanical treatment of the matter system and a quasi-classical approach for photonic modes—holding a balance between accuracy of the theoretical description of the system and the scalability of the method. In ref 259 a fully classical framework, cavity molecular dynamics (cavity-MD), is introduced which allows for simulations of large molecular systems in a cavity. Lastly, a number of related multimode methods have been investigated which incorporate different semiclassical ideas e.g., mapping to model systems.^252,260^

Future research focuses on realistic light–matter systems and ubiquitous quantum optical effects in real molecules under ambient conditions. The cMTE method integrates seamlessly with RT-TDDFT which paves the way for such investigations and ongoing work explores these possibilities.

### 14.4 Exact Factorization Approach

The mixed quantum-classical methods above involve approximating the force on the classical subsystem (nuclei in Ehrenfest and SH, and photons in cMTE). Instead, an exact force for a classical subsystem interacting with a quantum one,^261^ and corresponding exact equations for the quantum subsystem^262^ linked to the classical trajectories can be derived via the exact factorization. Computing the exact quantities driving the subsystem’s behavior even for small model systems can lead to insights into electron–nuclear-photon correlation and also in understanding errors arising from the approximate approaches. Pragmatically, the exact factorization provides a solid starting point to derive new practical mixed quantum-classical approximations.

While this is a general approach to coupled quantum subsystems, the application to polaritonic systems can proceed in a variety of ways, depending on which degrees of freedom are chosen as the marginal ones and which as conditional.^236^ Letting $\underline{\mathrm{R̲}},\underline{\mathrm{r̲}},\underline{\mathrm{q̲}}$ represent all nuclear, electronic, and photonic coordinates respectively), the works so far have explored the following factorizations of the full wave function:

109 $$\Psi \left(\underline{\mathrm{r̲}},\underline{\mathrm{R̲}},\underline{\mathrm{q̲}},t\right)\overset{228}{=}\chi \left(\underline{\mathrm{R̲}},t\right)\Phi_{\underline{\mathrm{R̲}}}\left(\underline{\mathrm{r̲}},\underline{\mathrm{q̲}},t\right)\overset{237}{=}\chi \left(\underline{\mathrm{r̲}},t\right)\Phi_{\underline{\mathrm{r̲}}}\left(\underline{\mathrm{R̲}},\underline{\mathrm{q̲}},t\right)\overset{261}{=}\chi \left(\underline{\mathrm{q̲}},t\right)\Phi_{\underline{\mathrm{q̲}}}\left(\underline{\mathrm{r̲}},\underline{\mathrm{R̲}},t\right)\overset{229}{=}\chi \left(\underline{\mathrm{R̲}},\underline{\mathrm{q̲}},t\right)\Phi_{\underline{\mathrm{R̲}},\underline{\mathrm{q̲}}}\left(\underline{\mathrm{r̲}},t\right)$$

where, a key reference is indicated above each equality sign. In each case, the conditional probability integrates to unity, e.g., for the first one shown $\int drdq|\Phi_{\underline{\mathrm{R̲}}}\left(\underline{\mathrm{r̲}},\underline{\mathrm{q̲}},t\right){|}^{2}=1$ . The terms in both the equation of motion for the marginal and the conditional contain exactly the effect of coupling to the other subsystems, and each depends on the solution of the other equation, so they must be solved self-consistently. While any choice is equivalent and in principle contains the complete information about the electron–photon-nuclear correlation, which degree of freedom is of primary interest in a given situation guides the choice in a given situation. The marginal satisfies a time-dependent Schrödinger equation with a scalar and vector potential, while the Hamiltonian for the conditional factor is more complicated.^233,234^ The potentials in the equation for the marginal system can therefore be interpreted directly and comparisons made with traditional approximate approaches; further, they provide exact forces for a classical treatment of that degree of freedom, yielding a rigorous starting point for developing mixed quantum-classical methods, as has been emphasized and advanced in the cavity-free molecular dynamics case.^261,263–267^ Coherence and decoherence effects emerge naturally in these approaches, unlike in surface-hopping and Ehrenfest, where ad hoc adjustments need to be made.

Before discussing what has been explored so far with each of the factorizations in eq 109, we note that it is equally exact to instead consider a Born–Huang-type of expansion corresponding to each partitioning choice. In particular, the first equality would correspond to $\Psi \left(\underline{\mathrm{r̲}},\underline{\mathrm{R̲}},\underline{\mathrm{q̲}},t\right)=\sum_{n}\chi_{n}\left(\underline{\mathrm{R̲}},t\right)\Phi_{\underline{\mathrm{R̲}}}^{n}\left(\underline{\mathrm{r̲}},\underline{\mathrm{q̲}}\right)$ , where $\Phi_{\underline{\mathrm{R̲}}}^{n}\left(\underline{\mathrm{r̲}},\underline{\mathrm{q̲}}\right)$ are the eigenstates of the polaritonic Hamiltonian $H_{\mathrm{pol}}\left(\underline{\mathrm{R̲}}\right)=H^{\mathrm{PF}}-\hat{T_{n}\;}$ in which the nuclear coordinates appear as parameters and the eigenvalues $\epsilon \left(\underline{\mathrm{R̲}}\right)$ give the polaritonic surfaces $\epsilon \left(\underline{\mathrm{R̲}}\right)$ .^228,229^ This framework has provided significant insight into cavity-modified chemistry, with distortions of the polaritonic surfaces away from the cavity-free Born–Oppenheimer ones indicating regions of strong electron–photon coupling. Nonadiabatic couplings between surfaces are however essential for cavity-modified chemistry to be predicted in any quantitative sense, and one would need to solve a system of many coupled equations for the $\chi_{n}\left(\underline{\mathrm{R̲}},t\right)$ . Moreover, to solve any realistic system would require approximations, and in particular, for mixed quantum-classical approaches, no unique prescription follows from the expansion as to what the force on the classically treated nuclei should be (e.g., the Ehrenfest and surface-hopping approaches use forces at two extremes, in a sense). In contrast, the single product form of the exact factorization formulation leads naturally to a uniquely defined force on the nuclei when the Hamiltonian for the nuclear part in the first factorization is treated classically.^261^ Instead, the challenge here is then to derive accurate approximations with practical implementations for this force and for the other coupling terms, in particular because of the coupling of the trajectories that is an inherent feature of the approach.^261,263–267^ While not so far explored, one can write similar formally exact Born–Huang-type expansions corresponding to the other factorizations in eq 109. For example, doing so for the fourth partitioning relates to the cavity-Born–Oppenheimer (cavity-BO) states that had been introduced in ref 54: $\Psi \left(\underline{\mathrm{r̲}},\underline{\mathrm{R̲}},\underline{\mathrm{q̲}},t\right)=\sum_{n}\chi_{n}\left(\underline{\mathrm{R̲}},\underline{\mathrm{q̲}},t\right)\Phi_{\underline{\mathrm{R̲}},\underline{\mathrm{q̲}}}^{n}\left(\underline{\mathrm{r̲}}\right)$ , where $\Phi_{\underline{\mathrm{R̲}},\underline{\mathrm{q̲}}}^{n}\left(\underline{\mathrm{r̲}}\right)$ are eigenstates of *H* ^*PF*^ – *T̂*_*n*_ – *T̂*_*p*_, where ${\mathrm{T̂}}_{p}=\frac{1}{2}\sum_{\alpha }{\mathrm{p̂}}_{\alpha }^{2}$ , i.e., the photonic degrees of freedom join the nuclear ones of the usual Born–Oppenheimer approach in parametrizing a Hamiltonian for the electronic subsystem, and the eigenvalues define the cavity-BO surfaces.

The factorization represented by the first equality yields an exact time-dependent potential energy surface for the nuclei, when coupled to electrons and photons. Unlike the polaritonic surfaces, this time-dependent surface has features that correlate directly with the evolving nuclear dynamics, as was shown for a model of cavity-induced suppression of proton-coupled electron transfer triggered by an initial photoexcitation.^226,268^ This surface itself distinguishes situations where the system begins already in a polaritonic state as would happen if the initial excitation is done in the presence of the cavity, or in a factorized photon-matter state, as would happen if the molecule was electronically excited and then placed in the cavity, and forces derived from it naturally lead to the dynamics of branching of the nuclear wavepacket into regions of different polaritonic character. The exact-factorization based mixed quantum-classical approximations performed well on this system,^269^ and while they have yet to be applied to more realistic polaritonic systems, their performance on cavity-free photoexcited molecular dynamics suggests they could offer improvements on the usual surface-hopping and Ehrenfest approaches.

On the other hand, the exact potential driving electronic motion was studied using the second factorization in eq 109, where step and peak features were found to play a seminal role in polaritonic squeezing of the electronic states and in photon-assisted delocalization.^235^ This choice of factorization is of particular interest to QEDFT (Section 5), since it provides an exact benchmark for the photon–electron correlation potential.

Turning now to the third factorization of eq 109, a study of the exact potential driving the photons in a simple model of a two-level electronic system coupled to a resonant photon mode, revealed that a barrier feature associated with photon emission is crucial in leading to an accurately predicted intensity.^257^ The lack of this feature in the cMTE approach could explain the underestimation of photon number and intensities observed in that approach.^251,252,255^ Building such a feature into an improved mixed quantum-classical approximation would be of great interest and has yet to be done. Simply applying the coupled-trajectory mixed quantum-classical scheme developed for the electron–nuclear problem^263,264,267^ does not capture these features since that algorithm was derived for a large mass ratio between the marginal and conditional coordinate, which is not the case when the photon displacement coordinate is chosen as the marginal. This was verified in ref 227 where the fourth factorization of eq 109 was applied, including now both the nuclear and photonic coordinates into the marginal. The exact time-dependent surface in this fourth factorization is in a sense an exactification of the cavity-BO concept,^54^ based on separating the electronic time-scale from the nuclear and photonic one.

The exploration of exact-factorization based methods for polaritonic systems is still at a young stage, and further development and testing is required, but the indications are that it would be well-worth pursuing to overcome deficiencies in surface-hopping and Ehrenfest methods while remaining practically feasible, and to shed light on properties of electron–photon correlation functionals of QEDFT.

## 15 Quantum Simulation of QED

While various many-body methods provide critical insight into molecular polaritonic systems, capturing their full quantum complexity—especially in the presence of large molecular ensembles, strong vibronic coupling, and nonequilibrium dynamics—quickly pushes classical algorithms beyond their practical limits.^54^ Quantum simulation emerges as a promising computational paradigm to overcome these limitations.^270^ By leveraging controllable quantum hardware and quantum algorithms tailored to polaritonic Hamiltonians, this approach offers a pathway to explore collective dynamics and cavity-modified reactivity that are presently inaccessible to classical computation.

Quantum hardware is particularly well suited to simulate the entangled, high-dimensional wave functions intrinsic to coupled light–matter systems. Recent work by Sheng and co-workers^271^ demonstrates a variational quantum algorithm (VQA) tailored to polariton chemistry, combining Fermionic encodings of molecules with variational boson encoders (VBE) for cavity photons and phonons. Their framework allows simulation of both Holstein–Tavis–Cummings (HTC) and Pauli–Fierz (PF) Hamiltonians on quantum simulators and superconducting hardware. Applied to a hydrogen molecule in a cavity, their approach achieved chemical accuracy after incorporating readout and reference-state error mitigation. Notably, their algorithm scales favorably, requiring only ∼ 22 qubits to simulate multimode cavity systems involving 20 molecules and 2 cavity modes, far beyond the tractable size for exact diagonalization.^271^

More broadly, quantum simulation strategies include both digital algorithms, such as Trotterized Hamiltonian evolution and qubitization, and hybrid schemes like the variational quantum eigensolver (VQE).^272,273^ Near-term VQE implementations have already demonstrated ground-state calculations for small cavity-coupled molecules using polaritonic unitary coupled cluster ansätze and qubit-efficient mappings.^274^ Extending these techniques to compute excitation spectra, response functions, or simulate real-time polariton dynamics will require algorithmic innovation and robust noise mitigation. Error correction remains a long-term goal, but effective error mitigation strategies, including those used by Sheng and co-workers, have already proven essential.

Realizing such simulations requires mapping key interactions, including electron–photon, phonon–exciton, and system–bath couplings, to quantum circuits. Circuit-QED platforms offer a natural analog, where superconducting qubits represent electronic degrees of freedom and microwave resonators encode cavity modes.^275^ Recent experiments have demonstrated fine-tuned control over light–matter coupling, including access to the ultrastrong regime.^276^ However, challenges remain in scaling, incorporating molecular vibrational structure, and simulating disorder or collective effects. Hybrid quantum–classical workflows remain crucial for near-term devices, including VQE, variational dynamics,^277^ and reduced-space encodings.

Ultimately, the goal is to enable application-driven simulations that address fundamental questions in polariton chemistry, such as how cavity fields modify reaction landscapes, tune nonadiabatic dynamics, or enable new quantum materials. As quantum hardware and algorithm design mature, quantum simulation promises to become an indispensable tool in the study of strongly coupled light–matter systems.

## 16 Outlook

The field of many-body QED has begun to illuminate new frontiers in quantum chemistry, where electronic, nuclear, and photonic degrees of freedom all contribute to the behavior of molecules in confined electromagnetic environments. This Perspective has reviewed some of the progress made toward developing accurate and scalable methods to describe such light–matter coupled systems from first principles. While the advances are promising, many challenges remain, and much of the theoretical landscape is still under active development.

Several directions appear particularly important. Continued refinement of exchange–correlation functionals in QEDFT will be necessary to capture anisotropic, multimode, and strong coupling regimes. Extensions that incorporate photon-mediated screening and collective effects, whether through diagrammatic, orbital-dependent, or data-driven approaches, may provide additional capabilities. In the context of QED-TDDFT, clarifying the assumptions underlying different approximations and developing improved frequency-dependent kernels could help address known limitations in excited-state and nonequilibrium response.

Wave function-based methods, such as QED-CASCI and QED-DMRG, provide rigorous access to strong correlation in polaritonic systems. However, further developments will be needed to handle large-scale configuration spaces that include nuclear, electronic, and photonic degrees of freedom. Similarly, QED-QMC approaches show significant promise, though additional work is required to address issues such as gauge ambiguities, bosonic basis convergence, and efficient sampling. Hybrid quantum–classical algorithms, including variational quantum eigensolvers with polaritonic ansätze, offer an emerging alternative for simulating QED systems on near-term quantum devices, although limitations in coherence, circuit depth, and qubit–mode mapping currently constrain their applicability.

We believe that many of the most compelling scientific questions in polaritonic chemistry, such as cavity-modified reactivity, collective energy transfer, or vibrational strong coupling, will benefit from deeper theoretical frameworks that combine QED with correlation, dynamics, and environment. Progress in this direction will likely require continued benchmarking, software innovation, and interdisciplinary collaboration across quantum chemistry, condensed matter physics, quantum optics, and quantum information. As a community, we are still in the early stages of building a predictive and transferable QED-based theory of light–matter chemistry. We hope that this growing body of theoretical work will continue to offer insight and inspiration as the field evolves.

## ◎

Physical Sciences Division, Pacific Northwest National Laboratory, Richland, Washington 99352, United States

## Notes

Author emails are as follows: Nicholas Bauman, nicholas.bauman@pnnl.gov; Leonardo A. Cunha, lcunha@flatironinstitute.org; A. Eugene DePrince, deprince@chem.fsu.edu; Johannes Flick, jflick@ccny.cuny.edu; Jonathan J. Foley, jfoley19@charlotte.edu; Gerrit Groenhof, gerrit.x.groenhof@jyu.fi; Norah Hoffmann, norah.hoffmann@nyu.edu; Karol Kowalski, karol.kowalski@pnnl.gov; Xiaosong Li, xsli@uw.edu; Marcus Liebenthal, marcus.liebenthal@pnnl.gov; Neepa J. Maitra, neepa.maitra@rutgers.edu; Ruby Manderna, rmandern@charlotte.edu; Mikuláš Matoušek, mikulda@volny.cz; Ilia M. Mazin, imazin@lanl.gov; Daniel Mejia-Rodriguez, daniel.mejia@pnnl.gov; Ajay Panyala, ajay.panyala@pnnl.gov; Bo Peng, peng398@pnnl.gov; Benjamin Peyton, peytonbe@msu.edu; Libor Veis, libor.veis@jh-inst.cas.cz; Nam Vu, nvu12@charlotte.edu; Jared D. Weidman, weidma10@msu.edu; Angela K. Wilson, akwilson@msu.edu; Rhiannon A. Zarotiadis, raz6681@nyu.edu; Yu Zhang, zhy@lanl.gov.
